# Effects of multiple conformers per compound upon 3-D similarity search and bioassay data analysis

**DOI:** 10.1186/1758-2946-4-28

**Published:** 2012-11-07

**Authors:** Sunghwan Kim, Evan E Bolton, Stephen H Bryant

**Affiliations:** 1 National Center for Biotechnology Information, National Library of Medicine, National Institutes of Health, Department of Health and Human Services, 8600 Rockville Pike, Bethesda, 20894, MD, USA

## Abstract

**Background:**

To improve the utility of PubChem, a public repository containing biological activities of small molecules, the PubChem3D project adds computationally-derived three-dimensional (3-D) descriptions to the small-molecule records contained in the PubChem Compound database and provides various search and analysis tools that exploit 3-D molecular similarity. Therefore, the efficient use of PubChem3D resources requires an understanding of the statistical and biological meaning of computed 3-D molecular similarity scores between molecules.

**Results:**

The present study investigated effects of employing multiple conformers per compound upon the 3-D similarity scores between ten thousand randomly selected biologically-tested compounds (10-K set) and between non-inactive compounds in a given biological assay (156-K set). When the “best-conformer-pair” approach, in which a 3-D similarity score between two compounds is represented by the greatest similarity score among all possible conformer pairs arising from a compound pair, was employed with ten diverse conformers per compound, the average 3-D similarity scores for the 10-K set increased by 0.11, 0.09, 0.15, 0.16, 0.07, and 0.18 for *ST*^*ST-opt*^, *CT*^*ST-opt*^, *ComboT*^*ST-opt*^, *ST*^*CT-opt*^, *CT*^*CT-opt*^, and *ComboT*^*CT-opt*^, respectively, relative to the corresponding averages computed using a single conformer per compound. Interestingly, the best-conformer-pair approach also increased the average 3-D similarity scores for the non-inactive–non-inactive (NN) pairs for a given assay, by comparable amounts to those for the random compound pairs, although some assays showed a pronounced increase in the per-assay NN-pair 3-D similarity scores, compared to the average increase for the random compound pairs.

**Conclusion:**

These results suggest that the use of ten diverse conformers per compound in PubChem bioassay data analysis using 3-D molecular similarity is not expected to increase the separation of non-inactive from random and inactive spaces “on average”, although some assays show a noticeable separation between the non-inactive and random spaces when multiple conformers are used for each compound. The present study is a critical next step to understand effects of conformational diversity of the molecules upon the 3-D molecular similarity and its application to biological activity data analysis in PubChem. The results of this study may be helpful to build search and analysis tools that exploit 3-D molecular similarity between compounds archived in PubChem and other molecular libraries in a more efficient way.

## Background

PubChem [1-4] is a public repository for biological activities of small molecules, consisting of three primary databases: PubChem Substance, PubChem Compound, and PubChem BioAssay. The PubChem Substance database (record identifier: SID) archives chemical information provided by individual data depositors, and the PubChem Compound database (record identifier: CID) contains the unique chemical structure contents extracted from the PubChem Substance database. Biological testing results of small molecules are archived in the PubChem BioAssay database (record identifier: AID). PubChem is a sizeable system with more than 92 million substance descriptions, 32 million unique small molecules, 620 thousand biological assays, and 170 million result outcomes (results from a substance tested in an assay is a result outcome). For efficient use of this enormous amount of information, PubChem provides various search and analysis tools to assist users in locating desired information.

The PubChem3D project [5-11] augments the utility of PubChem, by adding computed three-dimensional (3-D) descriptions to about 90% of the small molecules contained in the PubChem Compound database [6,11]. Each of these may include multiple 3-D conformations that are sampled to remove redundancy, guaranteeing a minimum (non-hydrogen atom pair-wise) root-mean-square distance (RMSD) between conformers. In addition, a diverse conformer ordering gives a maximal description of the conformational diversity of a molecule when using only a subset of sampled conformers [8,11]. A pre-computed search per compound record (called “Similar Conformers”) [8,11] gives immediate access to a set of 3-D similar compounds in PubChem and their respective superpositions. Systematic augmentation of PubChem resources to include a computed 3-D similarity layer grants users new capabilities to search, subset, visualize, analyze, and download data [11].

A goal of PubChem3D is to build a publicly accessible platform for virtual screening and biological activity analysis that exploits 3-D molecular similarity. However, there are many issues to address to achieve this goal, as discussed in a recent review by Sutherland *et al*. [12] One of the issues is that there is no obvious answer to what similarity threshold value should be used to determine whether two molecules are structurally similar. Our previous study [10] attempted to address this question in part, by investigating the distributions of the Rapid Overlay of Chemical Structures (ROCS) [13-21]-based 3-D similarity scores used in PubChem3D [8,10,11] between 270 billion unique compound pairs arising from 734,485 biologically tested compounds (referred to as the 734-K set hereafter) using a single conformer per compound. [See the Methods section for the definition of six 3-D similarity score types used in PubChem3D.] These distributions allow one to perform a statistical significance test that considers the null hypothesis that a particular similarity score between two molecules occurs by chance. The 3-D similarity score matrices generated were used to investigate structural differences between “non-inactives” and “inactives” for each of 1,389 bioassays archived in the PubChem BioAssay database at the time. [Note that the term “non-inactive” is defined as anything not specified to be “inactive” by the PubChem depositor and is used in place of “active”, since the definition of an “active” is not always specified in PubChem and many “non-inactives” are indeed “active”. More detailed explanation about the use of the non-inactives is given in the Methods section]. Although some PubChem assays showed a very clear structural separation between the non-inactives and the inactives in terms of 3-D similarity, the overall average similarity score for non-inactive–non-inactive (NN) pairs was found to be very similar to that for non-inactive–inactive (NI) pairs, indicating minimal or no difference between the NN and NI pairs in terms of 3-D similarity in general.

Although the previous study [10] provides an important statistical guideline for 3-D similarity search used in PubChem, there is still much room for improvement. For example, the previous study employed a single conformer per compound, which may not be sufficient for reliable evaluation of 3-D similarity between compounds, as the choice of a different conformer may yield substantially different similarity values and makes the selection of an appropriate conformer a significant consideration. An important characteristic of 3-D similarity methods, compared to 2-D similarity methods, is that 3-D similarity methods are applied at a *conformer* level, not at a *compound* level, to enable consideration of various distinct molecular conformations in 3-D space that may be biologically relevant. This suggests that, for 3-D similarity methods to provide biologically meaningful results, the conformer generation program employed should be able to routinely reproduce known “bioactive” conformers (*e.g.,* an experimentally-derived ligand conformation of a chemical bound in a protein crystal structure binding pocket). Indeed, many strategies have been developed for high-quality computational prediction of bioactive conformation of molecules [6,13,22-28]. A common approach to bioactive conformer generation is to sample energetically-accessible representatives that cover the biologically-accessible conformational space of a molecule. In general, the count of potentially relevant bioactive conformers increases as a function of molecular size and flexibility, making the count of conformers in a conformer ensemble an important factor to determine the quality of the ensemble; as the greater the count, the greater the probability of finding a representative conformer sufficiently similar to a relevant bioactive conformer of a molecule. In the case of PubChem3D, each 3-D conformer model consists of up to 500 conformers (with an average of 115 conformers) [11]. However, for tractability reasons, many PubChem3D services allow only up to *ten* “diverse” conformers per compound per request [11]. Note that a diverse conformer ordering gives a maximal description of the conformational diversity of a molecule when only a subset of available conformers is used [11].

Although many studies have investigated on the quality of various conformer model generators [13,22,28-31], a relatively few studies have dealt with the effects of the size of the conformer models upon the ligand-based 3-D virtual screening and biological activity analysis [15,32,33]. Recently, Kirchmair *et al.*[32] examined the impact of the quality of conformer models upon the hit list from pharmacophore-based and shape-based 3-D virtual screenings against four different protein targets: cyclin-dependent kinase 2 (CDK2), p38 mitogen-activated protein (MAP) kinase, peroxisome proliferator-activated receptor γ (PPAR-γ), and Factor Xa. It was found that, whereas the pharmacophore-based screening using CATALYST [34,35] was able to identify the greatest number of known actives even with very small conformer ensembles, the shape-based screening using ROCS showed an increased accuracy with larger conformer models. On the contrary, Hawkins *et al.*[15] reported that the ROCS-based virtual screening using a single conformation for a query molecule outperforms a pharmacophore modeling using the pharmacophores developed from multiple active compounds (up to 20). In addition, the same study [15] also reported that the performance of ROCS was not affected by whether a computationally-generated low-energy conformer or experimentally determined protein-bound structure was used as a single-conformer query. In this regard, a study on pharmacophore-based 3-D searching by Fox *et al.*[33] is also noteworthy, which examined the effect of conformer sampling upon within- and between-class similarity across seven different pharmacological classes containing 88 compounds in total. Including more conformations in pharmacophore multiplet bitmaps was found to increase both the within-class and between-class similarities, the net result being that the ratio between the two falls off as more and more conformations are included in the calculations. Overall, there is no consensus on the effects of the size of the conformer models upon ligand-based 3-D virtual screening and biological activity analysis and how many conformations should be considered in general.

The present study investigates effects of employing multiple diverse conformers per compound upon 3-D similarity computation (often referred to here as “multiple-conformer effects”) in two parts. The first part examines the question: how will employing multiple conformers per compound affect the 3-D similarity score between two randomly selected biologically-tested compounds? In the second part of the study, an attempt is made to answer the question: can one find a greater separation between inactives and non-inactives in PubChem bioassays on average when multiple conformers are used for each compound? Given that it is beyond our computational means to rigorously examine this question using all PubChem3D conformers, the approach used to tackle this second question involves constructing per-assay distributions of 3-D similarity scores for NN pairs using both a single conformer per compound and ten diverse conformers per compound and comparing them with equivalent results for random compound pairs of biologically tested compounds. In addition, multiple-conformer effects upon the separation between the non-inactive and inactive spaces are inferred based on the results of this study.

## Results

### Definitions and notations

In the present study, 3-D similarity computations that employ a single conformer per compound and multiple diverse conformers per compound are referred to as the “single-conformer approach” and the “multiple-conformer approach”, respectively. The multiple-conformer approach is further classified into two different approaches: the “best-conformer-pair” approach and the “all-conformer-pair” approach. In the best-conformer-pair approach, a similarity score between a single conformer and a compound (or between two compounds), where each compound has multiple diverse conformations, is represented by the greatest similarity score among all conformer pairs considered per conformer-compound pair (or compound-compound pair). In the all-conformer-pair approach, one may treat each of the individual conformer pairs as if it were a unique compound pair. These two different methods for the multiple-conformer approach were employed to help simulate different database search or analysis strategies using 3-D molecular similarity. The five different 3-D similarity usage scenarios considered in this study are summarized in Table 1.

**Table 1:** Different 3-D similarity search (or analysis) scenarios considered

**Search Scenario**	**Query**	**Conformer model**	**Description**
A	Compound	Single conformer	Similarity scores for a compound “query” compared to those of the “database” compounds, both computed using a single conformer per compound.
B	Compound	Multiple conformer, All-conformer-pair approach	Similarity scores that one may expect when each “query” conformer is compared to a set of multiple diverse conformers of the “database” compounds, using the “all-conformer-pair” approach. That is, all unique conformer pairs contribute to the average and standard deviation of the resulting similarity scores.
C	Conformer	Multiple conformer, All-conformer-pair approach	Similarity scores that one may expect when a single “query” conformer is compared to a set of multiple diverse conformers of the “database” compounds, using the “all-conformer-pair” approach. That is, all unique conformer pairs contribute to the average and standard deviation of the resulting similarity scores.
D	Conformer	Multiple conformer, Best-conformer-pair approach	Similarity scores that one may expect when a single “query” conformer is compared to a set of multiple diverse conformers of the “database” compounds using the “best-conformer-pair” approach. That is, only the conformer pair with the greatest similarity per conformer-compound pair contributes to the average and standard deviation of the resulting similarity scores.
E	Compound	Multiple conformer, Best-conformer-pair approach	Similarity scores that one may expect when each “query” conformer is compared to a set of multiple diverse conformers of the “database” compounds using the “best-conformer-pair” approach. That is, only the conformer pair with the greatest similarity per compound-compound pair contributes to the average and standard deviation of the resulting similarity scores.

Five different conformer handling scenarios considered in this study, where the 3-D similarity “query” is the entity being compared to a “database” of compound conformers.

As described in the Methods section, the six different score types were considered: shape-Tanimoto (ST), color-Tanimoto (CT), and combo-Tanimoto (ComboT) for each of the ST- and CT-optimizations. For convenience, superscript “ST-opt” or “CT-opt” is used to indicate whether the similarity score is estimated in the ST-optimized alignment or in the CT-optimized alignment (*i.e.*, *ST*^*ST-opt*^, *CT*^*ST-opt*^, *ComboT*^*ST-opt*^, *ST*^*CT-opt*^, *CT*^*CT-opt*^, and *ComboT*^*CT-opt*^), and the similarity scores from the single-conformer and multiple-conformer approaches are denoted with subscripts “single” and “multi”, respectively. Similarly, subscripts “best” and “all” are used to indicate the best-conformer-pair approach and all-conformer-pair approach, respectively. For example, *ST*_*best*_^*CT*-*opt*^ represents the CT-optimized ST score using the best-conformer-pair approach and *CT*_*all*_^*ST*-*opt*^ indicates the ST-optimized CT score using the all-conformer-pair approach. *ComboT*_*single*_^*ST-opt*^ indicates the ST-optimized ComboT score from the single-conformer-per-compound model. The word “XT” is used when we refer to any of the similarity measures (*i.e.*, ST, CT, and ComboT), or to a similarity score in a general sense.

### Datasets

Two different compound datasets were used in the present study: the 10-K set and the 156-K set. The 10-K set contains 10,000 biologically tested compounds randomly selected from the 734-K set used in the previous study [10]. The 156-K set consists of 156,232 CIDs that had computationally derived 3-D conformer models available in PubChem3D *and* that were non-inactive in at least one bioassay archived in the PubChem Bioassay database (as of January 25, 2010). The construction of these datasets is described in more detail in the Methods section, and the PubChem Compound CIDs included in the two sets are available in Additional files 1 and 2. In Table 2, the 3-D molecular descriptors for the two datasets (10-K and 156-K) are compared with those of the 734-K set and the entire PubChem3D contents [10]. Considering the average and standard deviations of the molecular descriptors, the two datasets used in the present study have property profiles nearly identical to those in the previously studied 734-K set and the entire PubChem3D contents, with the average molecular property and first standard deviation of each property almost completely overlapping the other datasets.

**Table 2:** Summary statistics of chemical structure descriptors

	**10-K set**	**156-K set**	**734-K set**	**Entire PubChem3D Contents**
Heavy atom count	24.5 ± 6.4	25.1 ± 6.4	24.6 ± 6.4	26.3 ± 7.0
Rotatable bond count	5.5 ± 2.7	5.5 ± 2.8	5.5 ± 2.7	6.8 ± 3.0
Effective rotor count	6.1 ± 2.8	6.1 ± 2.9	6.1 ± 2.8	7.4 ± 3.0
RMSD^thresh^	0.8 ± 0.2	0.8 ± 0.2	0.8 ± 0.2	0.9 ± 0.3
Monopole volume (Å^3^)	475.4 ± 124.7	487.0 ± 123.3	474.1 ± 124.0	509.0 ± 137.1
Q_x_ (Å^5^)	13.8 ± 6.9	14.3 ± 7.2	12.6 ± 7.0	13.6 ± 7.8
Q_y_ (Å^5^)	3.5 ± 1.6	3.6 ± 1.6	3.3 ± 1.6	3.6 ± 1.8
Q_z_ (Å^5^)	1.4 ± 0.6	1.4 ± 0.6	1.3 ± 0.6	1.5 ± 0.6
Total feature count	8.1 ± 2.6	8.4 ± 2.7	8.1 ± 2.6	8.5 ± 2.7
Hydrogen- bond acceptor count	3.0 ± 1.6	2.9 ± 1.6	2.9 ± 1.6	3.0 ± 1.6
Hydrogen- bond donor count	1.1 ± 1.0	1.2 ± 1.0	1.1 ± 1.0	1.2 ± 1.0
Anion count	0.2 ± 0.4	0.2 ± 0.4	0.2 ± 0.4	0.2 ± 0.4
Cation count	0.6 ± 0.8	0.8 ± 0.9	0.6 ± 0.8	0.7 ± 0.9
Hydrophobe count	0.3 ± 0.6	0.3 ± 0.6	0.3 ± 0.6	0.5 ± 0.8
Ring count	3.0 ± 1.2	3.1 ± 1.2	3.0 ± 1.2	3.0 ± 1.3

The average and standard deviation of heavy atom count, rotatable bond count, effective rotor count, sampling RMSD (RMSD^thresh^), monopole volume, three steric shape quadrupole components (Q_x_, Q_y_, and Q_z_), and feature counts (by total and each of the six feature types) for 10,000 randomly selected biologically tested compounds (10-K set), 156,232 non-inactive compounds (156-K set), 734,486 CIDs biologically tested compounds (734-K set) and the entire PubChem3D contents (26,157,365 CIDs as of September 2010). The data for the 734-K set and the entire PubChem3D contents are from Ref. [10]. The RMSD^thresh^ and effective rotor count were computed using Equations (1) and (3), respectively [see the Methods section].

### Similarity scores for the randomly selected conformer pairs

**Figure 1:**
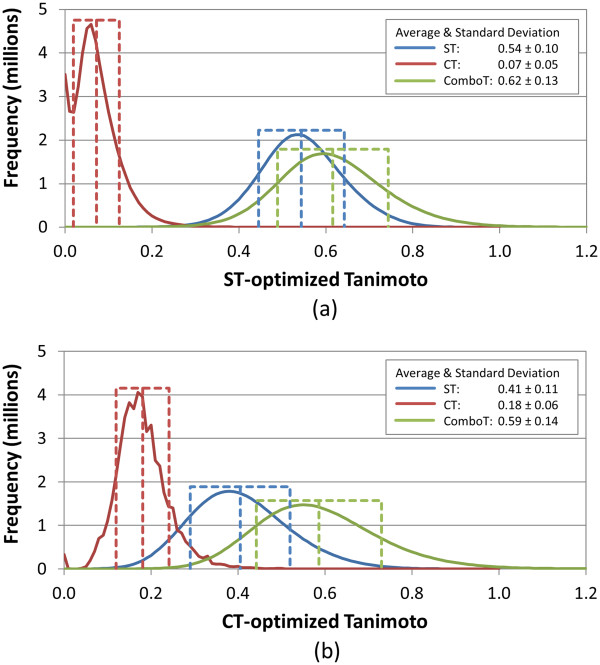
**Similarity distributions for “single-conformer” (*Scenario A*) approach.** Binned distributions in 0.01 increments of the 3-D similarity scores for the unique “conformer-conformer” pairs arising from 10,000 randomly selected biologically tested compounds (10-K set), computed using a single conformer per compound for **(a)** ST-optimized and **(b)** CT-optimized superpositions.

**Figure 2:**
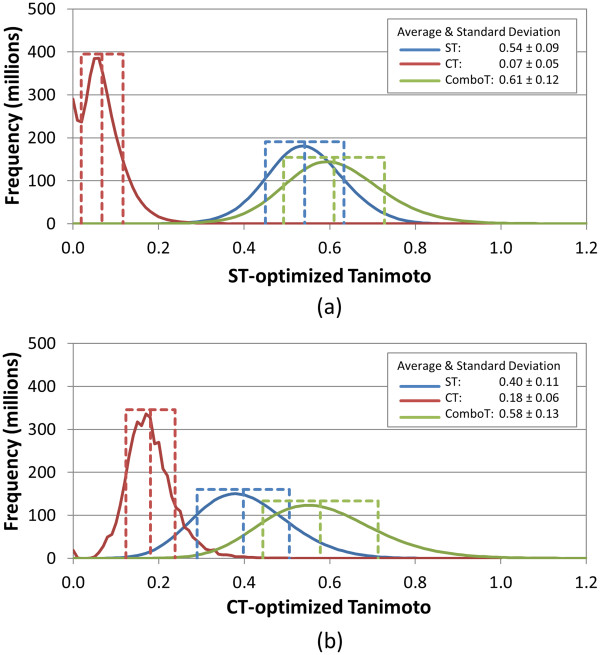
**Similarity distributions for multi-conformer “all-conformer-pair” (*Scenario B*) approach.** Binned distributions in 0.01 increments of the 3-D similarity scores for the unique “conformer-conformer” pairs arising from 10,000 randomly selected biologically tested compounds (10-K set), computed using ten diverse conformers per compound and the “all-conformer-pair” approach for **(a)** ST-optimized and **(b)** CT-optimized superpositions.

**Figure 3:**
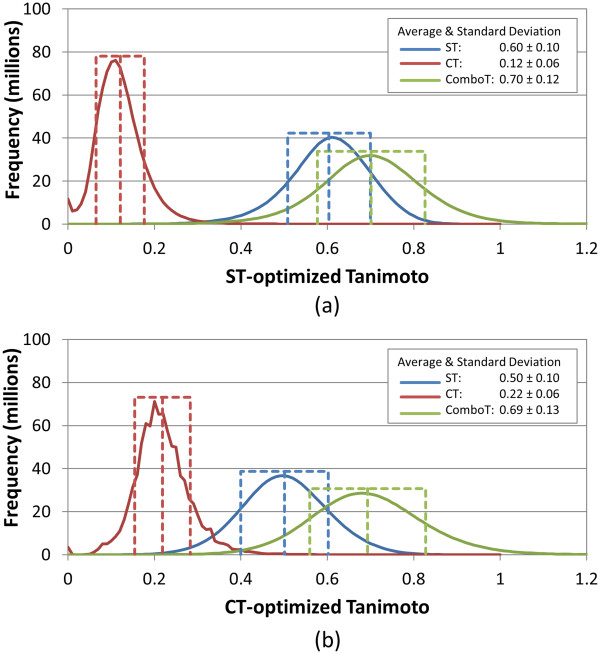
**Similarity distributions for multi-conformer “best-conformer-pair” (*Scenario D*) approach.** Binned distributions in 0.01 increments of the 3-D similarity scores for the unique “conformer-compound” pairs arising from 10,000 randomly selected biologically tested compounds (10-K set), computed using ten diverse conformers per compound and the “best-conformer-pair” approach for **(a)** ST-optimized and **(b)** CT-optimized superpositions.

**Figure 4:**
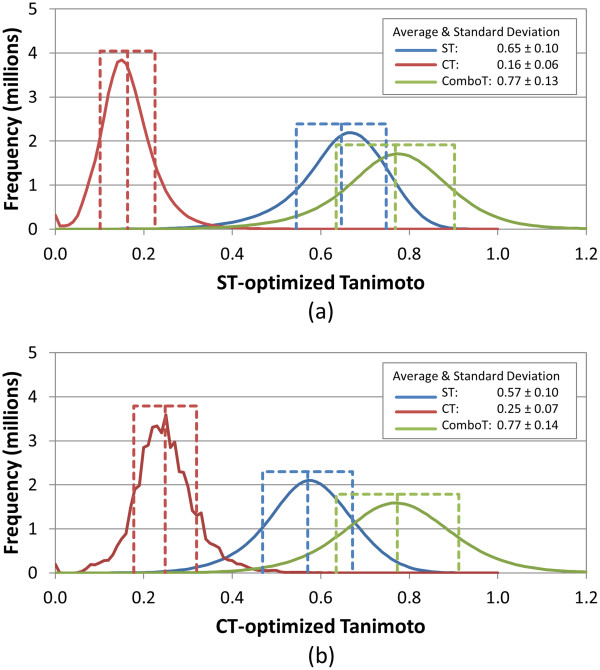
**Similarity distributions for multi-conformer “best-conformer-pair” (*Scenario E*) approach.** Binned distributions in 0.01 increments of the 3-D similarity scores for the unique “compound-compound” pairs arising from 10,000 randomly selected biologically tested compounds (10-K set), computed using ten diverse conformers per compound and the “best-conformer-pair” approach for **(a)** ST-optimized and **(b)** CT-optimized superpositions.

To investigate effects of employing multiple conformers per compound upon the 3-D similarity score between randomly selected biologically-tested compounds, the distributions of the 3-D similarity scores between the 10,000 compounds in the 10-K set were constructed using both the single-conformer and multiple-conformer approaches. The resulting 3-D similarity score distributions are shown in Figures 1, 2, 3, 4, and their averages and standard deviations are summarized in Table 3. For the single-conformer approach (Figure 1, corresponding to *Scenario A* in Table 1), the similarity score distributions for the unique conformer-conformer pairs and the unique compound-compound pairs are identical (since only one conformer per compound is considered). For the multiple-conformer approach, however, three different distributions were generated: the unique conformer-conformer pairs (Figure 2, *Scenario B*), the unique conformer-compound pairs (Figure 3, *Scenario D*), and the unique compound-compound pairs (Figure 4, *Scenario E*). Note that the 3-D similarity scores for the unique conformer-compound pairs (in Figure 3) and unique compound-compound pairs (in Figure 4) were computed using the “best-conformer-pair” approach, meaning that only the greatest similarity score was chosen from all relevant conformer pairs [*i.e.*, up to 10 (= 1 × 10) conformer-conformer pairs per conformer-compound pair and up to 100 (= 10 × 10) conformer-conformer pairs per compound-compound pair, because ten diverse conformers per compound were used].

**Table 3:** Similarity score distribution statistics for the random compound pairs

	**Search Scenario**^***b***^	**N**^***c***^	**ST**		**CT**		**ComboT**	
			**μ**	**σ**	**μ**	**σ**	**μ**	**σ**
**ST-optimized**								
compound- compound^*a*^	A	1	0.54	0.10	0.07	0.05	0.62	0.13
compound- compound	A	1	0.54	0.10	0.07	0.05	0.62	0.13
conformer- conformer	B	10	0.54	0.09	0.07	0.05	0.61	0.12
conformer- compound	D	10	0.60	0.10	0.12	0.06	0.70	0.12
compound- compound	E	10	0.65	0.10	0.16	0.06	0.77	0.13
**CT-optimized**								
compound- compound^*a*^	A	1	0.41	0.11	0.18	0.06	0.59	0.14
compound- compound	A	1	0.41	0.11	0.18	0.06	0.59	0.14
conformer- conformer	B	10	0.40	0.11	0.18	0.06	0.58	0.13
conformer- compound	D	10	0.50	0.10	0.22	0.06	0.69	0.13
compound- compound	E	10	0.57	0.10	0.25	0.07	0.77	0.14

The overall average and standard deviation of the 3-D similarity score distributions by search (or analysis) scenario. The 10-K set was used unless otherwise indicated.

^*a*^ For the 734-K set from Ref. [10].

^*b*^ See Table 1 for Search Scenario description.

^*c*^ N is the number of diverse conformers employed per compound.

As shown in Table 3 and Figure 1, when the single-conformer approach (*Scenario A*) was employed, the average similarity score for the “unique” compound-compound pairs from the 10-K set was 0.54, 0.07, 0.62, 0.41, 0.18, and 0.59 for *ST*^*ST-opt*^, *CT*^*ST-opt*^, *ComboT*^*ST-opt*^, *ST*^*CT-opt*^, *CT*^*CT-opt*^, and *ComboT*^*CT-opt*^, respectively. These averages for the 10-K set are exactly identical to those for the 734-K set determined from the previous study [10], reflecting the fact that 10-K set was constructed from random sampling of the 734-K set, and importantly suggesting that the 10-K set is representative of the 734-K set.

Perhaps surprising to some, the distributions (Figure 2) and statistics (Table 3) of the 3-D similarity scores from the “all-against-all” conformer comparison using multiple diverse conformers per compound (*Scenario B*) are essentially identical to those computed with a single conformer per compound (Figure 1), showing that the single-conformer and multiple-conformer “all-against-all” comparisons yield near identical random distributions. This suggests that the 3-D similarity distributions for random conformer pairs of biologically tested chemicals, whether using a single conformer or multiple conformers, is a general result. It also suggests that further analysis of the 10-K set may be a reasonable representation of a much larger bioactivity data set corpus and that conclusions drawn from the 10-K set may be applicable in a more general sense as the 10-K set represents the 734-K set and is possibly extensible to or may otherwise represent the analysis of all biologically tested compounds in PubChem.

Comparison of Figure 3 to Figure 1 is rather telling. If one uses a single conformer query against a multi-conformer database (*Scenario D*), as is often done in a similarity query of a 3-D database, *e.g.*, for virtual screening purposes, the average random distribution values increase by 0.06, 0.05, 0.08, 0.09, 0.04, and 0.10 for *ST*^*ST-opt*^, *CT*^*ST-opt*^, *ComboT*^*ST-opt*^, *ST*^*CT-opt*^, *CT*^*CT-opt*^, and *ComboT*^*CT-opt*^, respectively, as a result from picking the best conformer pair out of the maximum of ten diverse conformers considered per database compound. By comparing Figure 4 to Figure 3, one sees that, if a multi-conformer 3-D query is used against a multi-conformer 3-D database (*Scenario E*), there is a further increase over the results of *Scenario D* in that the average random distribution values increase by 0.05, 0.04, 0.07, 0.07, 0.03, and 0.08 for *ST*^*ST-opt*^, *CT*^*ST-opt*^, *ComboT*^*ST-opt*^, *ST*^*CT-opt*^, *CT*^*CT-opt*^, and *ComboT*^*CT-opt*^, respectively, as a result of an additional order of magnitude increase in diverse conformer pairs considered per compound query. One keen observation is that, as the conformer pair count considered per compound pair increases from 1 to a maximum of 100, the width of the distribution curves (*i.e.*, the variation of the similarity scores) does not change very much, whereas the location of the distribution curves (*i.e.,* the average of the similarity scores) does. Furthermore, the average similarity score differences between the potential maximums of 10 and 100 conformer pairs per compound pair (Figure 3*vs.* Figure 4) are smaller by 0.01-0.02 than those between 1 and a potential maximum of 10 conformer pairs per compound pair (Figure 1*vs.* Figure 3), indicating a decrease in the rate of the similarity score change as a function of the order of magnitude of the conformer pair count increase. [This observed reduction could also partially reflect an effective reduction in the average count of diverse conformer pairs per compound considered, because not every compound considered will have ten diverse conformers associated. However, considering the 10-K set averages 9.0 diverse conformers per compound, this effect should not be large but would be of increasing importance as the logarithmic count of diverse conformers per compound is further increased.] This reduction in the rate of the average similarity score increase as a function of the logarithm increase of conformer pairs suggests that the similarity score change will eventually plateau (*i.e.*, at some point, consideration of additional diverse conformers per compound will cease to change the distribution average). This log/linear behavior is similar to that observed in our earlier work [8], where a corresponding increase in the logarithmic number of conformers resulted in a linear increase of 3-D similarity neighbors. With that said, at ten diverse conformers per compound, there still seems to be additional room for further increases in the random distribution average if one was to consider using more diverse conformers per compound. It may be important to point out that, since PubChem samples conformers and then picks a diverse subset of these sampled conformers, if one was to use conformers without sampling or picking a non-diverse subset, there may be additional shifts or changes in these average random distributions.

Note that the *CT*^*ST-opt*^ distribution in Figure 1 has a second peak at *CT*^*ST-opt*^ = 0. This bimodality is related to the definition of the *CT* score. If none of the fictitious “feature” atoms used in the *CT* score are proximate, it will result in a zero or near-zero *CT* score. Whereas the CT-optimization maximizes the *CT* score, the ST-optimization ignores it. Considering the shift in the *CT*^*ST-opt*^ and *CT*^*CT-opt*^ distributions is 0.11, the compound pairs with *CT*^*CT-opt*^ < 0.11 would have negative *CT*^*ST-opt*^ scores, which is smaller than the smallest possible value of the CT score. [Note that the CT score ranges from 0 to 1 by definition.] This shift builds up the zero counts, thus forming a second peak at *CT*^*ST-opt*^ = 0.

**Figure 5:**
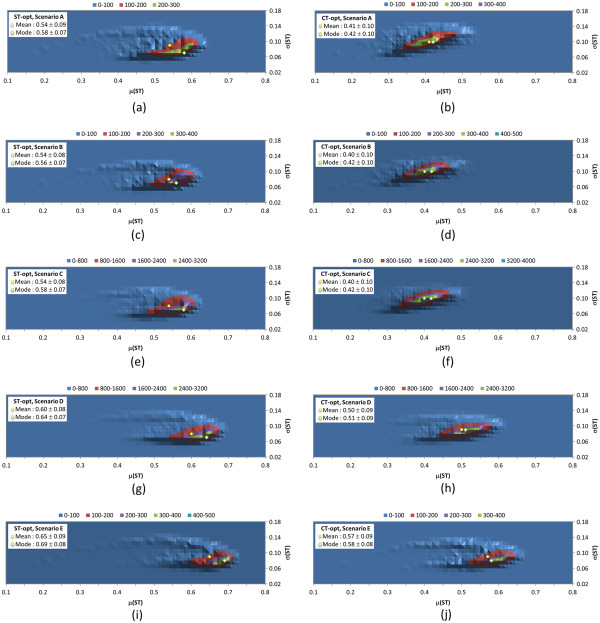
**Average and standard deviation distributions for shape-Tanimoto (ST), per “query”.** Binned distributions in 0.01 increments of the average and standard deviation of the shape-Tanimoto (ST) scores per query-type for the five similarity search scenarios tested (see Table 1): *Scenario A* [**(a)** and **(b)**], *Scenario B* [**(c)** and **(d)**], *Scenario C* [**(e)** and **(f)**], *Scenario D* [**(g)** and **(h)**], and *Scenario E* [**(i)** and **(j)**]. The left panels [**(a)**, **(c)**, **(e)**, **(g)**, and **(i)**] are for the ST-optimized ST scores, and the right panels [**(b)**, **(d)**, **(f)**, **(h)**, and **(j)**] are for the CT-optimized ST scores.

**Figure 6:**
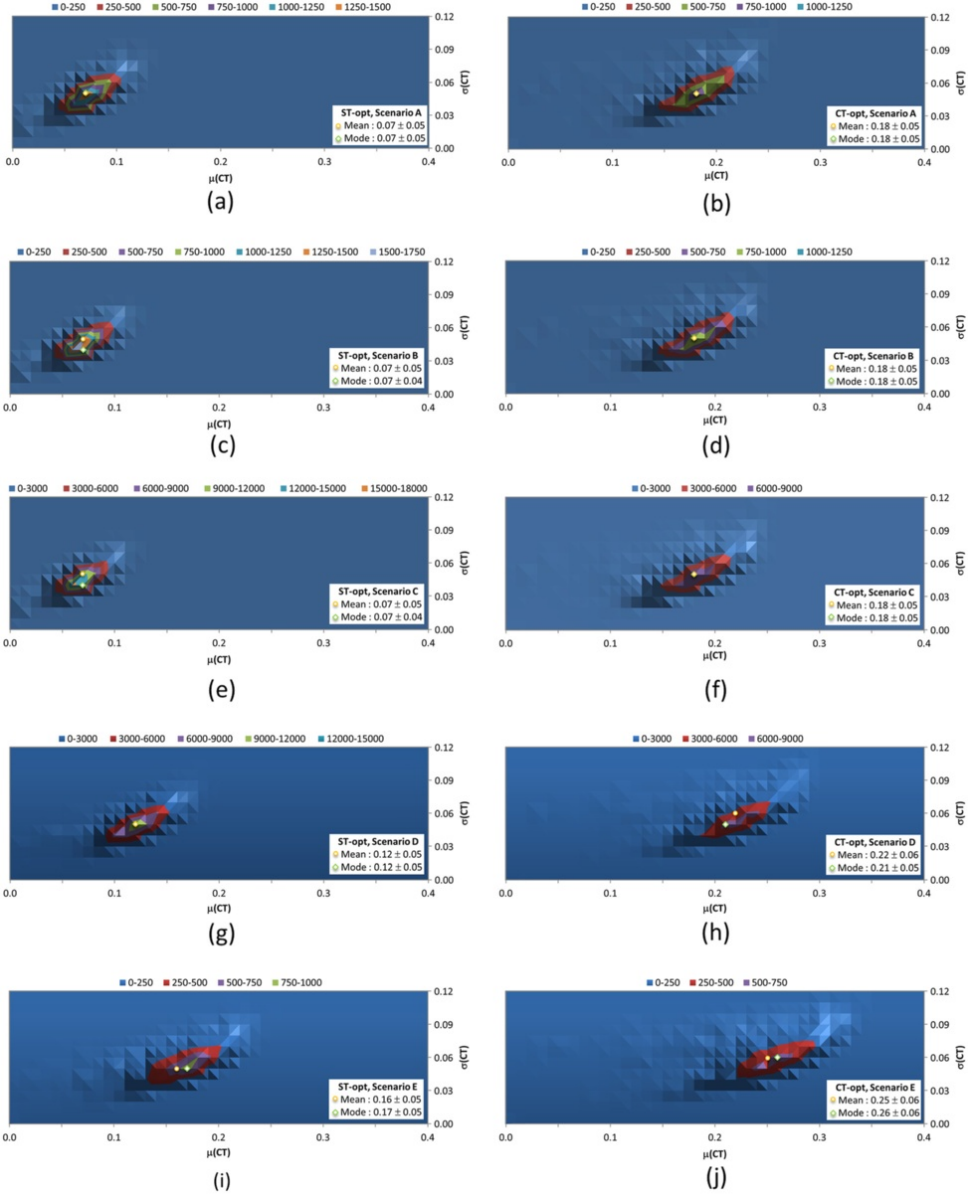
**Average and standard deviation distributions for color-Tanimoto (CT), per “query”.** Binned distributions in 0.01 increments of the average and standard deviation of the color-Tanimoto (CT) scores per query-type for the five similarity search scenarios tested (see Table 1): *Scenario A* [**(a)** and **(b)**], *Scenario B* [**(c)** and **(d)**], *Scenario C* [**(e)** and **(f)**], *Scenario D* [**(g)** and **(h)**], and *Scenario E* [**(i)** and **(j)**]. The left panels [**(a)**, **(c)**, **(e)**, **(g)**, and **(i)**] are for the ST-optimized CT scores, and the right panels [**(b)**, **(d)**, **(f)**, **(h)**, and **(j)**] are for the CT-optimized CT scores.

**Figure 7:**
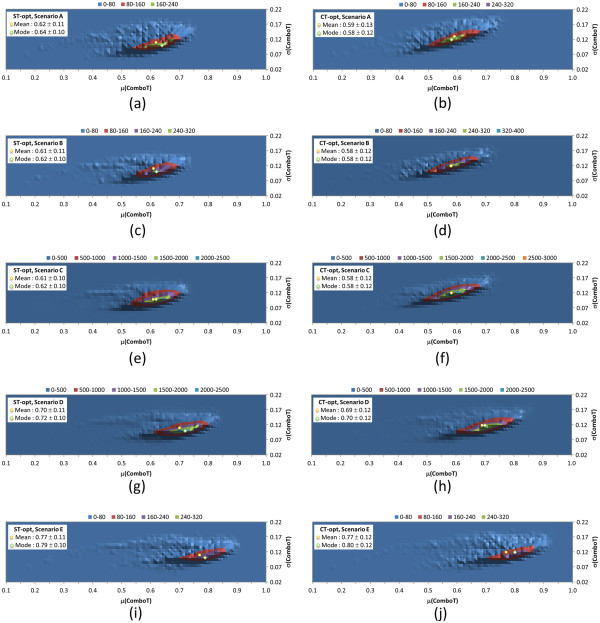
**Average and standard deviation distributions for combo-Tanimoto (ComboT), per “query”.** Binned distributions in 0.01 increments of the average and standard deviation of the combo-Tanimoto (ComboT) scores per query-type for the five similarity search scenarios tested (see Table 1): *Scenario A* [**(a)** and **(b)**], *Scenario B* [**(c)** and **(d)**], *Scenario C* [**(e)** and **(f)**], *Scenario D* [**(g)** and **(h)**], and *Scenario E* [**(i)** and **(j)**]. The left panels [**(a)**, **(c)**, **(e)**, **(g)**, and **(i)**] are for the ST-optimized ComboT scores, and the right panels [**(b)**, **(d)**, **(f)**, **(h)**, and **(j)**] are for the CT-optimized ComboT scores.

To further demonstrate what one might find in various 3-D similarity search/analysis scenarios, the similarity score matrices generated for the 10-K set were used to investigate the average and standard deviation of the “per-query” similarity scores for the five different scenarios described in Table 1. *Scenario A* uses a single conformer for each of the “query” and “database” compounds, and the other four search scenarios employ up to ten diverse conformers for each “database” compound. The “query” in *Scenario B* and *Scenario E* is a compound that may have up to ten diverse conformers whereas *Scenario C* and *Scenario D* use a single conformer as a “query”. *Scenario B* and *Scenario C* use the “all-conformer-pair” approach, while *Scenario D* and *Scenario E* use the “best-conformer-pair” approach. The resulting distributions from the five search scenarios are shown in Figures 5, 6, 7 for the ST, CT, and ComboT values, respectively.

Note that the “all-conformer-pair” approach effectively treats multiple conformers of a compound as individual compounds. For this reason, *Scenario B* and *Scenario C*, which adopt the all-conformer-pair approach, resulted in nearly identical average per-query similarity scores as *Scenario A*, which uses a single conformer per compound. These three search scenarios are conceptually identical to constructing the distribution curves for the unique compound-compound pair computed using the single-conformer approach (Figure 1) and those for the unique conformer-conformer pair computed using the “all-conformer-pair” approach (Figure 2). On the other hand, *Scenario D* and *Scenario E*, which use the “best-conformer-pair” approach, increased the per-query similarity scores. The averages for *Scenario D* and *Scenario E* were the same as those for conformer-compound pairs (Figure 3) and the unique compound-compound pairs (Figure 4), respectively, computed with multiple diverse conformers per compound.

The average per-query similarity scores in Figures 5, 6, 7 are nearly identical to the averages found in Table 3, but the standard deviations for the per-query similarity scores tend to be about 0.01 less than the standard deviations in Table 3. The modes of the average values are generally greater by 0.02-0.04 than the overall average values for most ST and ComboT values. Figures 5, 6, 7 suggest that some structures have smaller 3-D similarity search averages and greater standard deviations, yielding mode values that are shifted from the overall average values. This appears more pronounced in the case of ST-optimized similarity score values. So, depending on the mix of chemical structures being considered in an individual 3-D similarity search (and perhaps to the extent of their shape and feature uniqueness), there may be considerable volatility in the distribution of similarity scores between individual 3-D similarity queries. In the aggregate, however, most biologically considered chemicals in the 10-K set (and potentially PubChem in general) appear to have a limited range of variation in average 3-D similarity scores and standard deviation values.

### Similarity scores for the non-inactive–non-inactive pairs

#### A. Summary statistics

In the second part of this study, the distributions of the 3-D similarity scores between non-inactive compounds for each of the considered 1,528 bioassays archived in PubChem were constructed using the 156-K set and both the single-conformer and multiple-conformer approaches, to address the question: how will employing multiple conformers per compound change the 3-D similarity scores between the non-inactive molecules for a given biological assay? In addition, the results from this section, in conjunction with the analyses for the random compound pairs in the previous section, provide clues to the question: does one see (greater) separation of active and inactive spaces when employing multiple conformers per compound, as opposed to a single conformer per compound?

**Figure 8:**
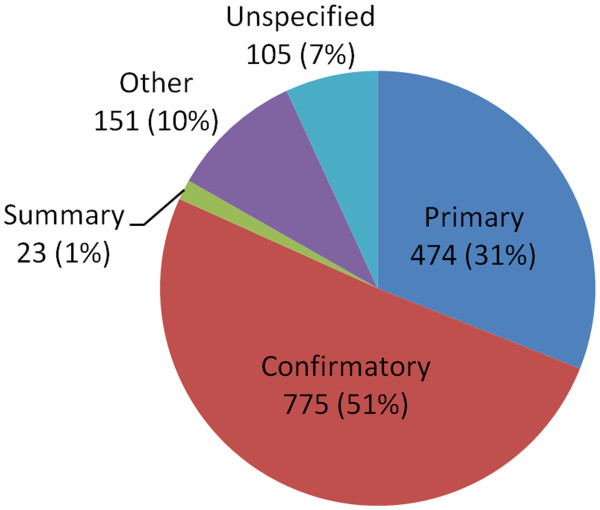
**Break down of assays by type.** Assay-type counts for the 1,528 bioassays considered in the present study.

**Figure 9:**
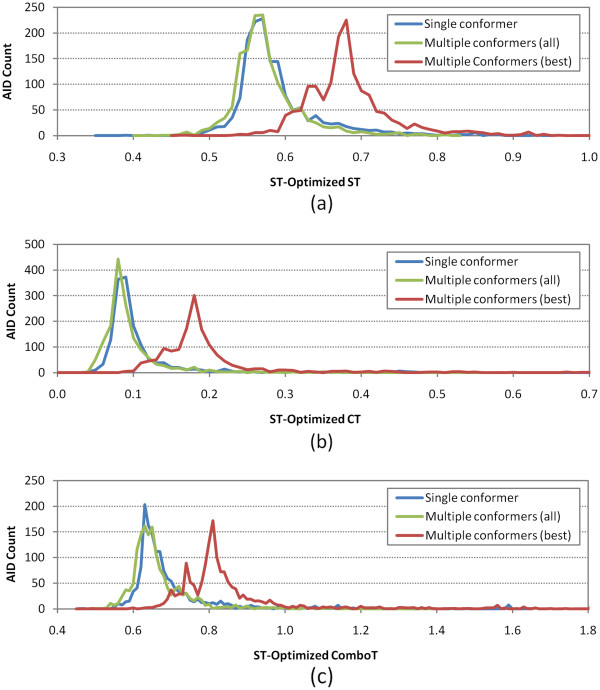
**Per-AID shape-Tanimoto (ST)-optimized 3-D similarity average values.** Binned distributions in 0.01 increments of the average 3-D similarity scores for non-inactive–non-inactive (NN) pairs of 1,528 AIDs in the PubChem BioAssay database, computed at the shape-Tanimoto-optimized superposition: **(a)** shape-Tanimoto (ST), **(b)** color-Tanimoto (CT), and **(c)** combo-Tanimoto (ComboT). “Single conformer”, “Multiple conformers (all)”, and “Multiple conformers (best)” correspond to search scenarios *A*, *B*, and *E*, respectively (See Table 1).

**Figure 10:**
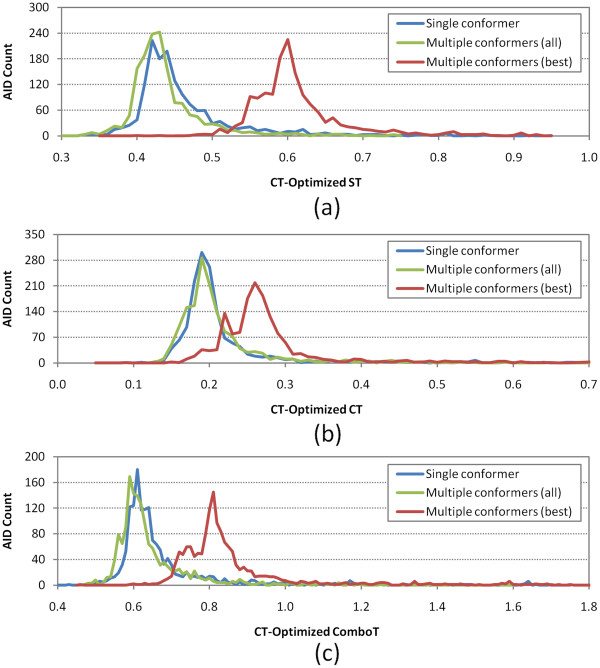
**Per-AID color-Tanimoto (CT)-optimized 3-D similarity average values.** Binned distributions in 0.01 increments of the average 3-D similarity scores for non-inactive–non-inactive (NN) pairs of 1,528 AIDs in the PubChem BioAssay database, computed at color-Tanimoto-optimized superposition: **(a)** shape-Tanimoto (ST), **(b)** color-Tanimoto (CT), and **(c)** combo-Tanimoto (ComboT). “Single conformer”, “Multiple conformers (all)”, and “Multiple conformers (best)” correspond to search scenarios *A*, *B*, and *E*, respectively (See Table 1).

The assay-type counts for these 1,528 bioassays are shown in Figure 8. The bioassays in the PubChem BioAssay database can be classified into four categories, according to PubChem depositor-assigned assay types: primary, confirmatory, summary, and other. Note that there is another category in Figure 8, “Unspecified”, because the assay-type attribute for AID records are optional, and not required. The per-AID average and standard deviation of the six 3-D similarity scores (*i.e.*, *ST*^*ST-opt*^, *CT*^*ST-opt*^, *ComboT*^*ST-opt*^, *ST*^*CT-opt*^, *CT*^*CT-opt*^, and *ComboT*^*CT-opt*^) for the NN pairs of each of the 1,528 AIDs are included in Additional file 3, and their overall per-AID average and standard deviation (*i.e.*, *μ*[*μ*(*XT*)], *σ*[*μ*(*XT*)], *μ*[*σ*(*XT*)] and *σ*[*σ*(*XT*)]; see the Methods section for the definition) are listed in Table 4 and Table 5. The average and standard deviation of the differences in these per-AID values between the single-conformer and multiple-conformer approaches are summarized in Table 6. The distributions of the per-AID average similarity scores for the 1,528 AIDs are shown in Figures 9 and 10.

**Table 4:** Summary statistics for per-AID shape-Tanimoto (ST)-optimized 3-D similarity

**Assay type**	**ST**^***ST-opt***^			**CT**^***ST-opt***^			**ComboT**^***ST-opt***^		
	**Single**	**All**	**Best**	**Single**	**All**	**Best**	**Single**	**All**	**Best**
**μ[μ(XT)]**									
Primary	0.58	0.57	0.68	0.10	0.09	0.20	0.68	0.65	0.84
Confirmatory	0.59	0.57	0.68	0.12	0.10	0.21	0.71	0.67	0.86
Summary	0.63	0.60	0.72	0.23	0.18	0.34	0.86	0.78	1.02
Other	0.58	0.57	0.67	0.11	0.08	0.21	0.68	0.65	0.84
Unspecified	0.56	0.56	0.62	0.07	0.07	0.13	0.63	0.62	0.73
All assays	0.58	0.57	0.68	0.11	0.09	0.20	0.70	0.66	0.85
*Random*	*0.54*	*0.54*	*0.65*	*0.07*	*0.07*	*0.16*	*0.62*	*0.61*	*0.77*
**σ[μ(XT)]**									
Primary	0.04	0.03	0.04	0.06	0.03	0.09	0.09	0.05	0.12
Confirmatory	0.06	0.05	0.06	0.11	0.05	0.13	0.16	0.09	0.18
Summary	0.11	0.09	0.09	0.20	0.15	0.21	0.31	0.24	0.30
Other	0.06	0.05	0.06	0.07	0.04	0.12	0.12	0.08	0.18
Unspecified	0.04	0.04	0.04	0.03	0.02	0.03	0.06	0.05	0.06
All assays	0.06	0.04	0.06	0.09	0.05	0.11	0.14	0.09	0.17
**μ[σ(XT)]**									
Primary	0.10	0.09	0.09	0.08	0.07	0.08	0.15	0.14	0.14
Confirmatory	0.11	0.10	0.10	0.10	0.09	0.11	0.19	0.16	0.18
Summary	0.11	0.09	0.09	0.16	0.13	0.15	0.25	0.20	0.22
Other	0.11	0.10	0.11	0.10	0.08	0.11	0.19	0.16	0.19
Unspecified	0.14	0.13	0.15	0.09	0.08	0.10	0.20	0.18	0.21
All assays	0.11	0.10	0.10	0.10	0.08	0.10	0.18	0.16	0.18
*Random*	*0.10*	*0.09*	*0.10*	*0.05*	*0.05*	*0.06*	*0.13*	*0.12*	*0.13*
**σ[σ(XT)]**									
Primary	0.02	0.01	0.01	0.05	0.03	0.04	0.07	0.03	0.05
Confirmatory	0.02	0.02	0.03	0.06	0.04	0.06	0.08	0.05	0.07
Summary	0.04	0.01	0.03	0.10	0.06	0.08	0.14	0.06	0.10
Other	0.03	0.02	0.03	0.06	0.04	0.05	0.07	0.04	0.07
Unspecified	0.02	0.02	0.03	0.03	0.03	0.04	0.04	0.04	0.05
All assays	0.03	0.02	0.03	0.06	0.04	0.05	0.08	0.05	0.07

The overall average and standard deviation of the AID-specific average and standard deviation as a function of search scenario and per assay type classifier. “All assays” corresponds to all assays irrespective of assay type. “*Random*” corresponds to the 10-K set results found from Table 3 as a means of comparison. “Single”, “All”, and “Best” correspond to search scenarios “A”, “B”, and “E” in Table 1, respectively.

**Table 5:** Summary statistics for per-AID color-Tanimoto (CT)-optimized 3-D similarity

**Assay type**	**ST**^***CT-opt***^			**CT**^***CT-opt***^			**ComboT**^***CT-opt***^		
	**Single**	**All**	**Best**	**Single**	**All**	**Best**	**Single**	**All**	**Best**
**μ[μ(XT)]**									
Primary	0.45	0.43	0.61	0.21	0.20	0.27	0.65	0.63	0.84
Confirmatory	0.47	0.44	0.61	0.22	0.21	0.29	0.69	0.65	0.87
Summary	0.52	0.49	0.66	0.32	0.31	0.40	0.84	0.79	1.03
Other	0.45	0.43	0.60	0.22	0.20	0.29	0.67	0.64	0.85
Unspecified	0.44	0.43	0.56	0.17	0.18	0.22	0.61	0.60	0.74
All assays	0.46	0.43	0.61	0.22	0.21	0.28	0.67	0.64	0.85
*Random*	*0.41*	*0.40*	*0.57*	*0.18*	*0.18*	*0.25*	*0.59*	*0.58*	*0.77*
**σ[μ(XT)]**									
Primary	0.05	0.04	0.05	0.06	0.04	0.08	0.10	0.06	0.13
Confirmatory	0.08	0.05	0.07	0.11	0.07	0.12	0.18	0.12	0.19
Summary	0.15	0.12	0.11	0.20	0.18	0.20	0.34	0.29	0.32
Other	0.07	0.06	0.07	0.08	0.06	0.12	0.14	0.11	0.19
Unspecified	0.04	0.03	0.04	0.03	0.03	0.03	0.07	0.06	0.07
All assays	0.07	0.05	0.07	0.09	0.06	0.11	0.16	0.11	0.17
**μ[σ(XT)]**									
Primary	0.13	0.11	0.10	0.08	0.08	0.08	0.18	0.16	0.15
Confirmatory	0.14	0.12	0.11	0.10	0.10	0.11	0.21	0.19	0.19
Summary	0.15	0.12	0.10	0.14	0.12	0.14	0.27	0.22	0.23
Other	0.14	0.12	0.12	0.11	0.10	0.11	0.21	0.18	0.20
Unspecified	0.15	0.14	0.15	0.11	0.12	0.12	0.21	0.21	0.22
All assays	0.13	0.12	0.11	0.10	0.09	0.10	0.20	0.18	0.18
*Random*	*0.11*	*0.11*	*0.10*	*0.06*	*0.06*	*0.07*	*0.14*	*0.13*	*0.14*
**σ[σ(XT)]**									
Primary	0.02	0.01	0.02	0.04	0.03	0.03	0.06	0.04	0.05
Confirmatory	0.03	0.02	0.03	0.05	0.04	0.05	0.08	0.05	0.07
Summary	0.06	0.01	0.03	0.09	0.04	0.07	0.15	0.05	0.11
Other	0.03	0.02	0.03	0.05	0.04	0.05	0.07	0.05	0.07
Unspecified	0.02	0.02	0.02	0.03	0.04	0.03	0.05	0.05	0.05
All assays	0.03	0.02	0.03	0.05	0.04	0.05	0.07	0.05	0.07

The overall average and standard deviation of the AID-specific average and standard deviation as a function of search scenario and per assay type classifier. “All assays” corresponds to all assays irrespective of assay type. “*Random*” corresponds to the 10-K set results found from Table 3 as a means of comparison. “Single”, “All”, and “Best” correspond to search scenarios “A”, “B”, and “E” in Table 1, respectively.

**Table 6:** Comparison of summary statistics of per-AID 3-D similarity

**Assay type**	**ST-optimized**						**CT-optimized**					
	**Best – Single**			**All – Single**			**Best – Single**			**All – Single**		
	**ST**	**CT**	**ComboT**	**ST**	**CT**	**ComboT**	**ST**	**CT**	**ComboT**	**ST**	**CT**	**ComboT**
**μ[μ(XT)]**												
Primary	0.10	0.10	0.16	-0.01	-0.01	-0.03	0.17	0.07	0.19	-0.02	-0.01	-0.03
Confirmatory	0.09	0.09	0.15	-0.02	-0.03	-0.04	0.15	0.07	0.18	-0.03	-0.01	-0.04
Summary	0.09	0.10	0.17	-0.03	-0.05	-0.08	0.14	0.08	0.19	-0.03	-0.02	-0.05
Other	0.09	0.10	0.16	-0.01	-0.02	-0.03	0.15	0.07	0.18	-0.02	-0.01	-0.04
Unspecified	0.06	0.06	0.10	0.00	-0.01	-0.01	0.11	0.04	0.12	-0.02	0.00	-0.01
All assays	0.09	0.09	0.15	-0.01	-0.02	-0.03	0.15	0.07	0.18	-0.02	-0.01	-0.03
**σ[μ(XT)]**												
Primary	0.02	0.04	0.05	0.02	0.04	0.06	0.03	0.03	0.05	0.03	0.03	0.06
Confirmatory	0.03	0.04	0.06	0.04	0.08	0.12	0.04	0.03	0.06	0.05	0.06	0.11
Summary	0.04	0.04	0.06	0.04	0.08	0.12	0.05	0.04	0.07	0.05	0.06	0.11
Other	0.04	0.07	0.09	0.02	0.04	0.06	0.05	0.05	0.09	0.03	0.04	0.07
Unspecified	0.01	0.01	0.02	0.02	0.01	0.02	0.02	0.01	0.02	0.02	0.01	0.03
All assays	0.03	0.04	0.06	0.03	0.06	0.09	0.04	0.03	0.06	0.04	0.05	0.09
**μ[σ(XT)]**												
Primary	0.00	0.01	0.01	0.00	0.00	0.01	0.01	0.01	0.01	0.00	0.00	0.01
Confirmatory	0.01	0.01	0.02	0.01	0.01	0.01	0.01	0.01	0.02	0.01	0.01	0.01
Summary	0.02	0.04	0.06	0.02	0.03	0.04	0.03	0.03	0.06	0.03	0.03	0.05
Other	0.01	0.01	0.01	0.00	0.01	0.01	0.01	0.01	0.01	0.01	0.01	0.01
Unspecified	0.01	0.01	0.01	0.01	0.00	0.01	0.01	0.01	0.01	0.01	0.00	0.01
All assays	0.01	0.01	0.02	0.01	0.01	0.01	0.01	0.01	0.02	0.01	0.01	0.01
**σ[σ(XT)]**												
Primary	0.01	0.02	0.02	0.01	0.01	0.02	0.01	0.01	0.03	0.01	0.01	0.02
Confirmatory	0.01	0.02	0.03	0.01	0.02	0.03	0.02	0.02	0.04	0.01	0.02	0.03
Summary	0.03	0.05	0.08	0.02	0.04	0.07	0.04	0.04	0.08	0.03	0.04	0.07
Other	0.01	0.02	0.02	0.01	0.01	0.02	0.01	0.02	0.03	0.01	0.01	0.02
Unspecified	0.01	0.01	0.03	0.01	0.01	0.02	0.02	0.01	0.03	0.01	0.01	0.02
All assays	0.01	0.02	0.03	0.01	0.02	0.02	0.02	0.02	0.03	0.01	0.01	0.03

The overall average and standard deviation of the AID-specific average and standard deviation of the similarity score differences between the multiple-conformer model and single-conformer model approaches. “All assays” corresponds to all assays irrespective of type. “Single”, “All”, and “Best” correspond to search scenarios “A”, “B”, and “E” in Table 1, respectively.

As described in the Methods section, the same analyses were also performed for a subset of the 1,528 assays, which consists of 843 assays that have active compounds only (without any inconclusive or unspecified compounds), and the results are summarized in Additional file 4. Note the minor peaks in the distributions for the best-conformer-pair approach in Figures 9, 10. These peaks arise from the 34 National Institutes of Neurological Disorders and Strokes (NINDS) approved drug screenings, in which the same set of non-inactive compounds were tested against different targets. Although they are different assays, they do have the same set of non-inactive compounds, yielding the minor peaks in Figures 9, 10. Because these 34 assays are not included in the 843 assays, the resulting similarity score distribution curves from the 843 assays are closer to the normal distribution than those from the 1,528 assays. However, the two assay sets have very similar averages and standard deviations to each other, and hence the analysis and discussion below, which are based on the 1,528 assay set, also hold for the 843 assay set.

Summarized in Table 6, the overall average and standard deviation of the per-AID average similarity score differences between the best-conformer-pair approach and single-conformer approach were 0.09 ± 0.03, 0.09 ± 0.04, 0.15 ± 0.06, 0.15 ± 0.04, 0.07 ± 0.03, and 0.18 ± 0.06 for *μ*(*ST*_*best*−*single*_^*ST-opt*^), *μ*(*CT*_*best*−*single*_^*ST-opt*^), *μ*(*ComboT*_*best*−*single*_^*ST-opt*^), *μ*(*ST*_*best*−*single*_^*CT-opt*^), *μ*(*CT*_*best*−*single*_^*CT-opt*^), and *μ*(*ComboT*_*best*−*single*_^*CT-opt*^), respectively, indicating that the best-conformer-pair approach gives a statistically significant increase in 3-D similarity scores between the NN pairs, relative to those computed using a single conformer per compound. On the other hand, the overall averages and standard deviations for *μ*(*ST*_*all*−*single*_^*ST*-*opt*^), *μ*(*CT*_*all*−*single*_^*ST-opt*^), *μ*(*ComboT*_*all*−*single*_^*ST*-*opt*^), *μ*(*ST*_*all*−*single*_^*CT*-*opt*^), *μ*(*CT*_*all*−*single*_^*CT*-*opt*^), and *μ*(*ComboT*_*all*−*single*_^*CT*-*opt*^) were −0.01 ± 0.03, −0.02 ± 0.06, −0.03 ± 0.09, −0.02 ± 0.04, −0.01 ± 0.05, and −0.03 ± 0.09, respectively, meaning that there were no statistically significant differences in the average 3-D similarity scores for the NN pair between the all-conformer-pair approach (*Scenario B*) and the single-conformer approach (*Scenario A*).

In general, as shown in Tables 4 and 5, when going from primary screen assays to confirmatory assays to summary assays, the average similarity scores between the NN pairs increase, regardless of whether a single conformer or multiple conformers are used for each compound. However, these increases should not be considered as statistically meaningful because the standard deviations of the NN-pair 3-D similarity scores also become greater and these distributions significantly overlap.

Employing multiple conformers per compound (*Scenario E* as opposed to *Scenario A*) increases the NN-pair 3-D similarity scores by a similar amount for all of the primary, confirmatory, and summary assays. For example, the average and standard deviation of *μ*(*ComboT*_*best*−*single*_^*ST*-*opt*^) were 0.16 ± 0.05, 0.15 ± 0.06, and 0.17 ± 0.06, for primary, confirmatory, and summary assays, respectively (Table 6). Therefore, the multiple-conformer effects upon the 3-D similarity score of the NN pairs should be considered as independent of the assay category.

#### B. Comparison between the NN-pairs and randomly selected pairs

If one considers the data from Table 3 (*i.e.*, the rows labeled as “Random” in Table 4 and Table 5) and compares them to the per-AID results, one sees that for randomly selected biologically tested molecules the overall averages are consistently less than the per-AID values across all 3-D similarity optimization types and across both single-and multi-conformer approaches, with the notable exception of “Unspecified” assay types. This is a similar result found in the earlier study [10] that used a single conformer per compound.

**Figure 11:**
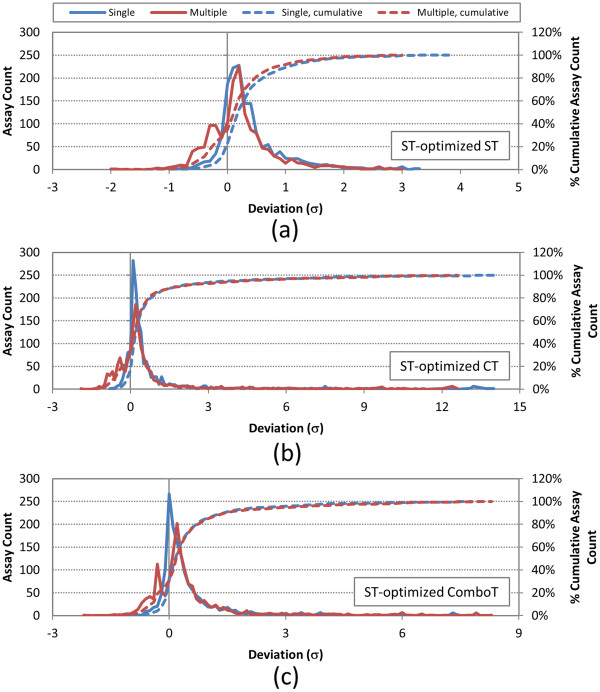
**Deviation from random of per-AID shape-Tanimoto (ST)-optimized 3-D similarity average values.** Deviation of the ST-optimized 3-D similarity scores for non-inactive–non-inactive (NN) pairs of 1,528 AIDs from the corresponding average for the random compound pairs, computed using both a single conformer and best multiple (ten) diverse conformers per compound: **(a)** ST-optimized ST, **(b)** ST-optimized CT, and **(c)** ST-optimized ComboT. The deviations are binned with increment of 0.1 standard deviation (*σ*) unit. “Single” and “Multiple” refer to search scenarios *A* and *E*, respectively (See Table 1).

**Figure 12:**
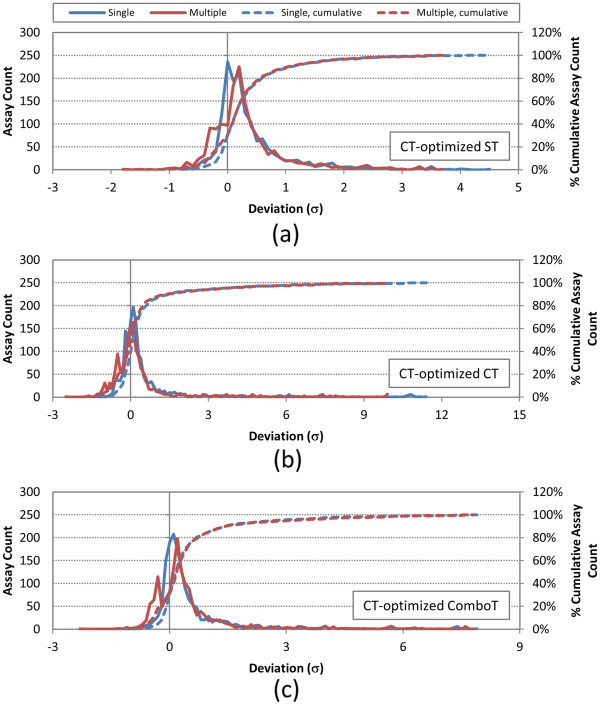
**Deviation from random of per-AID color-Tanimoto (CT)-optimized 3-D similarity average values.** Deviation of the CT-optimized 3-D similarity scores for non-inactive–non-inactive (NN) pairs of 1,528 AIDs from the corresponding average for the random compound pairs, computed using both a single conformer and best multiple (ten) diverse conformers per compound: **(a)** CT-optimized ST, **(b)** CT-optimized CT, and **(c)** CT-optimized ComboT. The deviations are binned with increment of 0.1 standard deviation (*σ*) unit. “Single” and “Multiple” refer to search scenarios *A* and *E*, respectively (See Table 1).

Table 7 and Figures 11 and 12 summarize how distant the average NN-pair similarity scores for each of the bioassays considered are from those for randomly selected compound pairs (from Table 3). Note that the per-AID NN-pair CT score average for a given assay are found as much as 14 standard deviation units away from the corresponding average for the random compound pairs, reflecting that the average and standard deviation of the CT scores for the random compound pair are less than those of the ST or ComboT scores.

**Table 7:** The cumulative count of biological assays whose non-inactive–non-inactive (NN) pairs have the average 3-D similarity score smaller than a given threshold

	**ST-optimized**						**CT-optimized**					
	**ST**		**CT**		**ComboT**		**ST**		**CT**		**ComboT**	
**Single conformer per compound**												
*μ* + *σ*	1339	(87.6)	1255	(82.1)	1289	(84.4)	1336	(87.4)	1309	(85.7)	1273	(83.3)
*μ* + 2*σ*	1490	(97.5)	1384	(90.6)	1425	(93.3)	1474	(96.5)	1410	(92.3)	1414	(92.5)
*μ* + 3*σ*	1517	(99.3)	1431	(93.7)	1460	(95.5)	1508	(98.7)	1440	(94.2)	1460	(95.5)
*μ* + 4*σ*	1528	(100.0)	1450	(94.9)	1494	(97.8)	1524	(99.7)	1463	(95.7)	1484	(97.1)
*μ* + 5*σ*	−	−	1468	(96.1)	1506	(98.6)	1528	(100.0)	1482	(97.0)	1503	(98.4)
*μ* + 6*σ*	−	−	1481	(96.9)	1514	(99.1)	−	−	1494	(97.8)	1511	(98.9)
*μ* + 7*σ*	−	−	1488	(97.4)	1517	(99.3)	−	−	1506	(98.6)	1516	(99.2)
*μ* + 8*σ*	−	−	1506	(98.6)	1528	(100.0)	−	−	1510	(98.8)	1528	(100.0)
*μ* + 9*σ*	−	−	1509	(98.8)	−	−	−	−	1516	(99.2)	−	−
*μ* + 10*σ*	−	−	1513	(99.0)	−	−	−	−	1519	(99.4)	−	−
*μ* + 11*σ*	−	−	1515	(99.1)	−	−	−	−	1526	(99.9)	−	−
*μ* + 12*σ*	−	−	1518	(99.3)	−	−	−	−	1528	(100.0)	−	−
*μ* + 13*σ*	−	−	1518	(99.3)	−	−	−	−	−	−	−	−
*μ* + 14*σ*	−	−	1527	(99.9)	−	−	−	−	−	−	−	−
*μ* + 15*σ*	−	−	1528	(100.0)	−	−	−	−	−	−	−	−
**Ten conformers per compound**												
*μ* + *σ*	1389	(90.9)	1281	(83.8)	1275	(83.4)	1349	(88.3)	1332	(87.2)	1280	(83.8)
*μ* + 2*σ*	1500	(98.2)	1375	(90.0)	1407	(92.1)	1473	(96.4)	1400	(91.6)	1409	(92.2)
*μ* + 3*σ*	1527	(99.9)	1415	(92.6)	1447	(94.7)	1514	(99.1)	1436	(94.0)	1445	(94.6)
*μ* + 4*σ*	1528	(100.0)	1438	(94.1)	1469	(96.1)	1528	(100.0)	1462	(95.7)	1470	(96.2)
*μ* + 5*σ*	−	−	1462	(95.7)	1490	(97.5)	−	−	1480	(96.9)	1493	(97.7)
*μ* + 6*σ*	−	−	1474	(96.5)	1503	(98.4)	−	−	1488	(97.4)	1510	(98.8)
*μ* + 7*σ*	−	−	1486	(97.3)	1519	(99.4)	−	−	1499	(98.1)	1521	(99.5)
*μ* + 8*σ*	−	−	1492	(97.6)	1527	(99.9)	−	−	1518	(99.3)	1528	(100.0)
*μ* + 9*σ*	−	−	1501	(98.2)	1528	(100.0)	−	−	1521	(99.5)	−	−
*μ* + 10*σ*	−	−	1515	(99.1)	−	−	−	−	1528	(100.0)	−	−
*μ* + 11*σ*	−	−	1521	(99.5)	−	−	−	−	−	−	−	−
μ + 12*σ*	−	−	1521	(99.5)	−	−	−	−	−	−	−	−
*μ* + 13*σ*	−	−	1528	(100.0)	−	−	−	−	−	−	−	−

Symbols *μ* and *σ* represent the average and standard deviation of the respective 3-D similarity scores between randomly selected compounds (from Table 3). Numbers in parentheses are the percent cumulative counts of biological assays. The single- and ten-conformer-per-compound values correspond to search scenarios “A” and “E”, respectively.

When averaged over the six different similarity score types, the single-conformer approach resulted in 1,279 AIDs (83.8%) with the NN-pair similarity scores equal to or greater than the corresponding average for the random compound pairs. The multiple-conformer approach reduced this number to 1,090 AIDs (71.4%) on average, implying a decrease in the distance of the NN-pair similarity from the random compound pair similarity in general. However, there is a minute difference between the ST scores and the CT and ComboT scores. When multiple conformers were used for each compound, there was a decrease in the difference between the ST scores of the NN-pairs and those of the random pairs for the entire range. On the other hand, as shown in Table 7, the multiple-conformer effect resulted in more bioassays that had NN-pair CT and ComboT score averages equal to or greater than the respective μ + 2σ thresholds. For example, when going from the single conformer per compound to ten diverse conformers per compound, the number of bioassays with *μ*(*CT*_*NN*-*pair*_^*ST*-*opt*^) ≥ *μ*(*CT*_*random*_^*ST-opt*^) + 2*σ*(*CT*_*random*_^*ST*-*opt*^) increases from 144 (= 1,528 − 1,384) to 153 (=1,528 − 1,375), whereas the number of bioassays with *μ*(*ST*_*NN*-*pair*_^*ST*-*opt*^) ≥ *μ*(*ST*_*random*_^*ST-opt*^) + 2*σ*(*ST*_*random*_^*ST-opt*^) decreases from 38 (=1,528 − 1,490) to 28 (=1,528 − 1,500).

#### C. Examples of multiple-conformer effects in 3-D similarity computation

This section presents examples that show substantial multiple-conformer effects upon 3-D similarity between biologically similar molecules. An underlying assumption of these examples is that a similarity score at least two standard deviations above the average similarity score of the randomly selected conformers (*i.e.*, greater than *μ* + 2*σ*) is statistically significant. For example, two compounds are considered to be structurally similar to each other when the *ComboT*^*ST-opt*^ score between them is greater than 0.88 and 1.03 for the single-conformer and best-conformer-pair approaches, respectively (on the basis of the statistical parameters in Table 3).

According to our supplementary computation, the average and standard deviation of the 2-D similarity scores between all compound pairs arising from the 10-K set, computed using the PubChem subgraph fingerprint [36] and Tanimoto equation [37-40], were 0.42 ± 0.13, and hence, a pair of molecules with the 2-D similarity score greater than 0.68 were considered to be structurally similar to each other under the same threshold (*i.e.*, *μ* + 2*σ*) as used for 3-D similarity. Note that 2-D similarity methods do not always recognize structural similarity between biologically similar molecules that 3-D similarity methods readily do [8,10,11,14,41-44].

In the examples below, each conformer of a given compound will be designated with a local conformer identifier (LID) [11], which, in conjunction with CID, allows the user to uniquely identify each conformer in PubChem3D. For simplicity, a particular conformer of a compound is represented by combining the corresponding CID and LID. For example, conformer “60823.2” represents LID 2 of CID 60823, the default conformer of atorvastatin. The default conformer of a compound record in PubChem3D is the first diverse conformer, which is used when a single conformer is considered for a molecule. Note that LID 1 of a compound is not necessarily the default conformer, because the diverse conformer ordering of a compound may or may not begin with LID 1.

**Figure 13:**
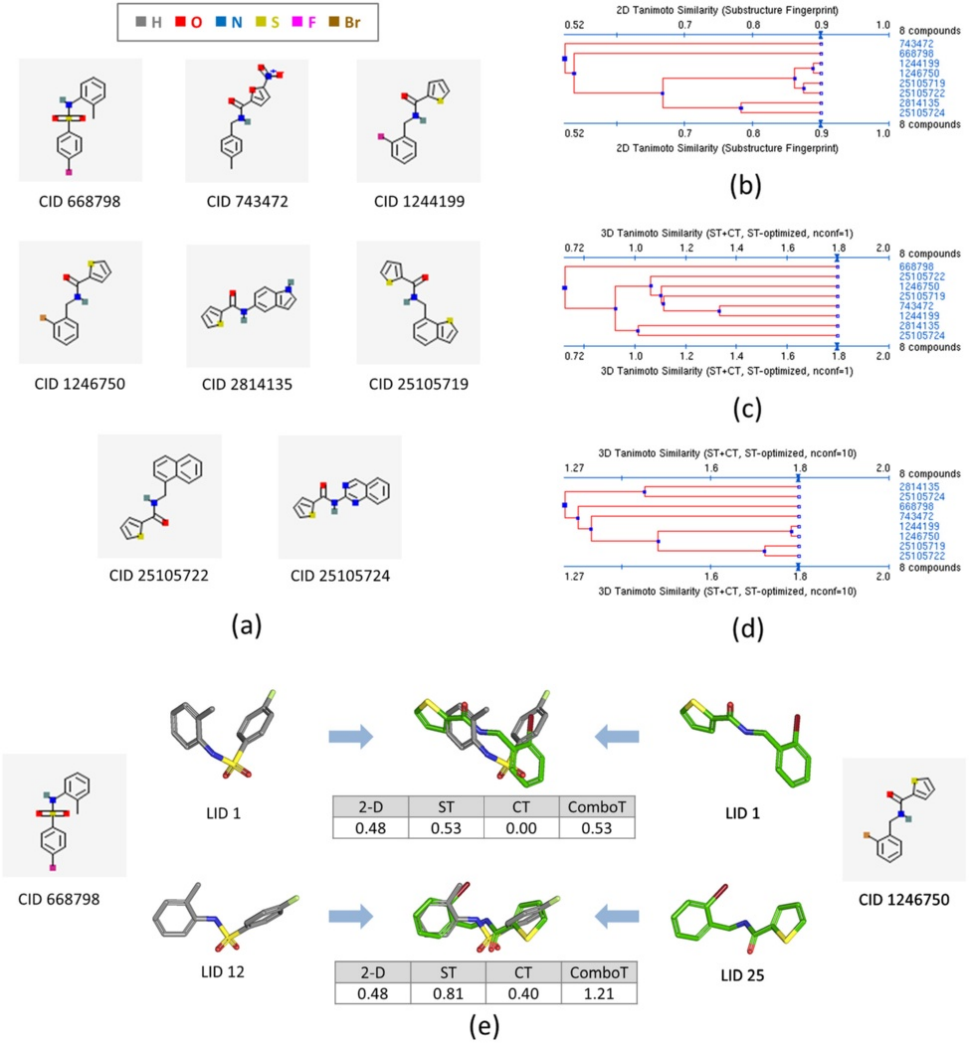
**Demonstrated multi-conformer effects using AID 1033.** Effects of employing multiple conformers per compound upon 3-D similarity of the non-inactive compounds tested in AID 1033. Eight compounds in panel **(a)** are non-inactive in AID 1033. Panel **(b)** depicts the dendrogram that shows the 2-D similarity among the eight structures, computed using the PubChem subgraph fingerprints. The dendrograms for the 3-D shape-optimized combo-Tanimoto (*ComboT*^*ST-opt*^) similarity are shown in panels **(c)** and **(d)** for a single conformer per compound and ten diverse conformers per compound, respectively. Panel **(e)** compares conformer superpositions between two of the non-inactive compounds (CIDs 668798 and 1246750). LID stands for the local identifier, which represents different conformers of a compound.

An example of substantial multiple-conformer effects upon 3-D similarity comparison can be found with the non-inactive compounds of AID 1033 [45] (Figure 13), an NMR-based screening to identify small molecules that target the chaperone DnaK in E.coli [46,47]. As shown in the dendrograms produced by the PubChem Structure Clustering tool [11] in Figure 13, whereas some compound pairs show 2-D similarity scores below 0.68, the 3-D *ComboT*^*ST-opt*^ similarity scores computed using ten conformers per compound are all well above 1.03. For example, the 2-D similarity score between CIDs 668798 and 1246750 is 0.48, and the *ComboT*^*ST-opt*^ score computed using a single default conformer is 0.53, implying that both the 2-D and single-conformer 3-D similarity cases cannot recognize structural similarity between the two molecules. However, when ten diverse conformers per compound are employed, the largest *ComboT*^*ST-opt*^ score from all the conformer pairs is 1.21 [corresponding to the (668798.12, 1246750.25) pair], sufficiently high enough to consider them structurally similar to each other.

**Figure 14:**
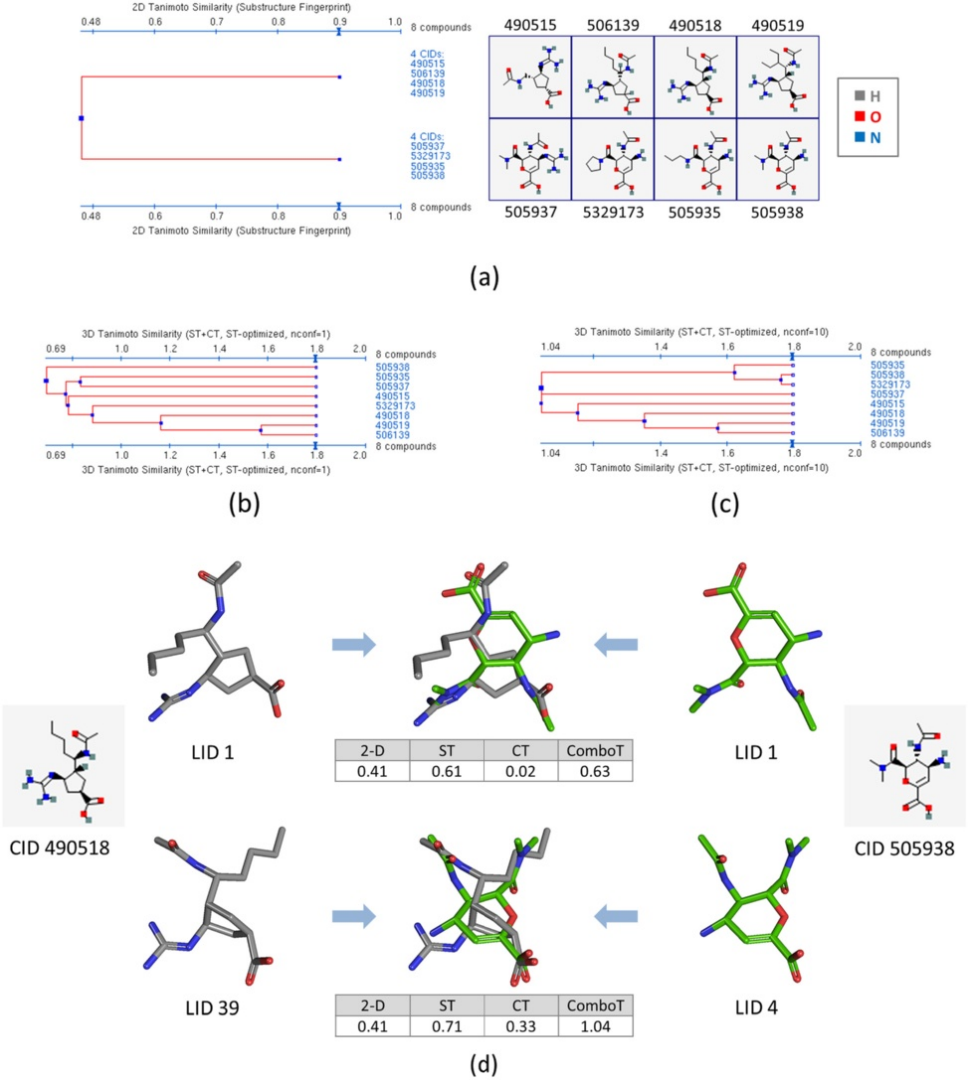
**Demonstrated multi-conformer effects using AID 491.** Effects of employing multiple conformers per compound upon 3-D similarity of non-inactive compounds tested in AID 491. Panel **(a)** shows the dendrogram based on 2-D similarity among eight compounds selected from 60 non-inactive compounds in AID 491. The dendrograms for the 3-D shape-optimized combo-Tanimoto (*ComboT*^*ST-opt*^) similarity are shown in panels **(b)** and **(c)** for a single conformer per compound and ten diverse conformers per compound, respectively. Panel **(d)** compares conformer superpositions between two of the non-inactive compounds (CIDs 490518 and 505938). LID stands for the local conformer identifier, which represents different conformers of a compound.

Another example in which the PubChem 3-D multi-conformer similarity method provides an improvement is AID 491 [48] (Figure 14), which contains in vitro affinity data extracted from the literature for small-molecule inhibitors tested against influenza A virus sialidase (also known as neuraminidase) [49,50]. Figure 14 shows the dendrograms for eight compounds selected from 60 non-inactive compounds in AID 491 for demonstration purposes. Although the eight compounds can be classified into two clusters of compounds at a 2-D similarity threshold of 0.5, the 3-D *ComboT*^*ST-opt*^ similarity among them is greater than 1.03 across all eight structures when ten conformers are used for each compound. In other words, the two independent 2-D similarity clusters, each representing a different chemical series, are recognized as a single 3-D similarity cluster, which in part emphasizes the relative strengths of the PubChem 3-D similarity method over its PubChem 2-D similarity counterpart. The 3-D similarity single-conformer approach, however, cannot recognize the similarity between all eight compounds. CIDs 490518 and 505938 are the compound pair that shows the greatest difference between the 2-D similarity score and the 3-D *CT*^*ST-opt*^ score (0.41 *vs.* 1.04). Note that the conformer superposition between 490518.39 and 505938.4 resulted in a substantial increase in the *CT*^*ST-opt*^ score, compared to the superposition between the default conformers 490518.1 and 505938.1.

#### D. Summary comparison of overall average similarity

**Figure 15:**
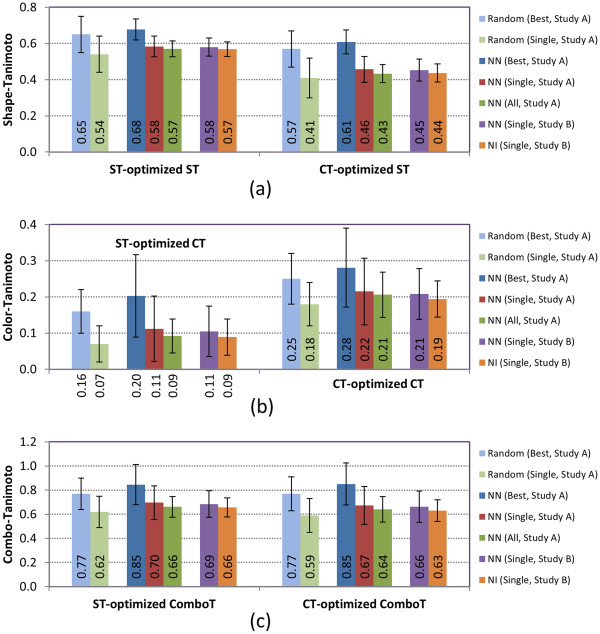
**Summary comparison of overall average similarity.** Comparison of the overall average 3-D similarity scores, *μμ*(*XT*)], for the non-inactive–non-inactive (NN) pairs with those for the non-inactive–inactive (NI) pairs and random compound pairs. The words, “Single”, “Best”, and “All”, in the legend box indicate the single-conformer approach (*Scenario A*), “best-conformer-pair” approach (*Scenario E*), and “all-conformer-pair” approach (*Scenario B*), respectively. Study A is the present study, and Study B is a previous study by Kim et al. (Ref. [10]).

Figure 15 compares the overall average 3-D similarity scores for the random compound-compound pairs with the overall average *μμ*(*XT*)] values for the NN and NI pairs, computed in the present and previous studies [10]. As shown in Figure 15, the single-conformer approach does not result in a noticeable difference between the average 3-D similarities for the NN pair and those for the random compound-compound pair, with distributions that considerably overlap. While there are individual assays where an improvement is found (*e.g.*, more AIDs with the average similarity of the NN pairs 2*σ* away from those of the random pairs in the case of CT and ComboT values), the use of the multiple-conformer approach does not make a noticeable improvement in the aggregate.

Why is this so? The lack of a more noticeable difference between the NN pairs and random pairs can be attributed to an assumption used in the molecular similarity methods and the nature of typical biological assays. All molecular similarity methods exploit the so-called similarity principle, which states that “structurally similar molecules are likely to have similar biological and pharmacological properties” [51]. An underlying assumption of the similarity principle is that structurally similar molecules tend to bind to a target macromolecule in a similar fashion. However, not all biological assays have a well-defined target macromolecule. For example, biological experiments may be designed to find molecules that target a whole cell or a whole organism, involving many different potential binding sites, modes of action, etc. Even when there is a well-defined target and carefully crafted assay, there is also no guarantee that the observed activity is real or manifested in an intended way, with the potential for molecules to bind irreversibly or otherwise denature the experiment by being cytotoxic, a chromophore at the detector wavelength, protein aggregator, etc. There is also no guarantee, after the activity observed is validated as being real, that the way in which two similar molecules bind will be identical (*e.g.*, agonist *vs*. antagonist *vs*. partial-agonist *vs*. partial-antagonist). Further confusing matters, there is no guarantee that the biologically inactive molecules are indeed not active for a given biological target with factors in how the assay is performed preventing or not registering such activity. The complications that one can imagine preventing accurate correlation of structural similarity with biological activity in one form or another are nearly limitless but one must try nevertheless to do the best they can with the data they have.

The 1,528 bioassays considered in this study were selected without considering any complexities, and therefore, there is no guarantee that the observed biological similarity between bioactive molecules in these assays arises from structural similarity. Without an assumption of correlation between structural and biological similarities for these bioactive molecules, expected structural similarity between bioactive molecules should not be very different from that between biologically (and structurally) unrelated molecules. This idea is consistent with the small difference in 3-D similarity scores between the NN-pairs and random compound-compound pairs, as depicted in Figure 15. In this context, the average similarity scores for the NI pairs should also be similar to those for the NN-pairs and the random pairs because the NI-pairs are biologically unrelated by nature, consistent with our previous study using a single conformer per compound (also compared in Figure 15). The multiple-conformer approach would not make any noticeable difference of the NI-pair from the NN pair and random pair “on average”, although the present study did not consider the 3-D similarity score computation of the NI pairs using the multiple-conformer approach, as its proper treatment would require a tremendous amount of additional computational resources beyond our current means.

## Discussion

An important question one may ask is: when you *randomly* select two compounds an *infinite* number of times, what does the distribution of their 3-D similarity scores look like? This distribution may be used to develop a statistical approach to test the null hypothesis that a particular similarity score between two compounds is likely to occur by chance. The distribution curves in Figures 1 and 4, generated from 49,995,000 compound pairs (arising from 10,000 randomly selected compounds), seem to be a good approximation for this purpose. Considering that the distribution curves from 270 billion compound pairs (arising from the 734-K compound set) for the single-conformer approach have very similar distribution curves to those in Figure 1 with identical averages and standard deviations (as summarized in Table 3), the distribution curves from an infinite number of random compound pairs from a chemical data set with a similar profile to PubChem’s is not likely to be very different from those in Figure 1. We believe that the 50 million compound pairs from the 10-K set are also enough for the multiple-conformer approach such that adding more compound pairs in the distributions in Figure 4 is not likely to change much. Therefore, we generated a conversion map from a 3-D similarity score between molecules to the *p*-value of getting that similarity score by chance, based on the distributions from Figures 1 and 4 for the single conformer and ten conformers per compound, respectively (Additional file 5). [These *p*-values were computed as 1 − percent cumulative distribution.]

There are two important factors that may affect the results of the present study: the choice of biological assays considered and the number of conformers used for each compound. For example, one may argue that a much clearer separation between the non-inactive and random spaces (or between “active” and “inactive” spaces) could have been observed if we considered assays with very well established pharmacology, using an increasing number of conformers per compound. While it is possible to do such analyses, it would be difficult to come up with a generalization from them. From our analyses, we already know that the per-AID NN-pair similarity score averages (Figures 9, 10) show a broad distribution; while some are very close to the average for the random compound pairs, others are much larger than that. This is because nearly every assay in PubChem is different from others in terms of various factors, not just underlying pharmacology or binding geometry. Therefore, even if we analyze some exceptional cases with a huge number of conformers per compound, we do not think it will be possible to extrapolate the results to a general case. Indeed, many studies [15,16,32,52-55] have tested various similarity methods using different data sets, typically taking a few well-defined protein targets and validated biological results, but no similarity method showed the same performance against different protein targets tested. Moreover, considering that a substantial number of PubChem biological assays do not even have a clear target protein, this “generalization from exception” does not seem very promising.

It is important to note that there is no general consensus on how many conformers are enough for 3-D similarity methods. As pointed out in a recent review by Scior *et al*. [12], to increase the reliability of the 3-D similarity comparison one must consider as many conformations per compound as possible to ensure adequate conformational space coverage. However, because it would require enormous computational resources, one should find a compromise between computational cost and sampling breadth. The choice of the number of conformers per compound in our study is directly related to the current capability and future direction of the PubChem services. While PubChem generates up to 500 conformers per compound, PubChem3D services provide support for up to ten (diverse) conformers per compound for scalability reasons and other considerations. The attempt here is to see if one can build on top of this limitation a virtual screening and biological data analysis platform with generic purpose. Therefore, the scope of the present study was determined based on this goal. While it is worth noting that it is possible to devise a set of PubChem assays that give definite clear separation of active and inactive space *even with a single conformer per compound*, as our previous study identified [10], the focus of the present study is on getting a big picture from very heterogeneous data in PubChem. The statistical profiles of 3-D similarity values for biologically tested compounds provided in this work will enable other analysis approaches, such as clustering of 3-D similarity values at statistically-appropriate thresholds, to identify useful subsets of non-inactive chemical structures involved in biological activity data trends, using a limited number of diverse conformers per compound.

## Conclusion

The present study investigated effects of using multiple conformers per compound upon the 3-D similarity values used by PubChem. In the first part of this study, the 3-D similarity score distribution curves were generated for the unique conformer-conformer, conformer-compound, and compound-compound pairs, using a single conformer and ten diverse conformers for each of 10,000 randomly selected compounds (Figures 1, 2, 3, 4 and Table 3). When each conformer was treated like a unique compound (*i.e.*, the “all-conformer-pair” approach), the all-against-all conformer comparison using ten diverse conformers per compound resulted in similarity score distributions nearly identical to those computed with a single conformer per compound. When similarity between two compounds was represented using the largest similarity score among possible conformer pairs arising from the two compounds (*i.e.*, the “best-conformer-pair” approach), the average similarity scores for randomly selected compounds increased as a function of logarithmic increase in the number of conformer pairs considered per compound pair. The best-conformer-pair approach with ten diverse conformers per compound resulted in the average random compound pair similarity score greater than those computed using the single-conformer approach, by 0.11, 0.09, 0.15, 0.16, 0.07, and 0.18 for *ST*^*ST-opt*^, *CT*^*ST-opt*^, *ComboT*^*ST-opt*^, *ST*^*CT-opt*^, *CT*^*CT-opt*^, and *ComboT*^*CT-opt*^, respectively.

Employing multiple conformers per compound also affected the average and standard deviation of the per-query similarity scores in a similar way (Figures 5, 6, 7). For example, while the all-conformer-pair approach (*Scenarios B* and *C*) resulted in nearly identical average per-query similarity scores to those from the single-conformer approach (*Scenario A*), the “best-conformer-pair” approach (*Scenario D* and *E*) increased the per-query similarity scores. The average per-query scores for *Scenarios A*, *B* and *C* were essentially identical to the average for randomly selected compound pair (Figure 1), computed using the single-conformer approach. The averages for *Scenario D* and *E* were the same as those for the unique conformer-compound pairs (Figure 3) and the unique compound-compound pairs (Figure 4), respectively, computed with multiple conformers per compound.

In the second part of this study, the distribution of the 3-D similarity scores for the NN pairs (Figures 9 and 10) was constructed for each of 1,528 bioassays archived in PubChem, using the 156-K set and both the single-conformer and multiple-conformers per compound. Whereas the average NN-pair 3-D similarity scores from the all-conformer-pair and single-conformer approaches did not differ very much from each other, the best-conformer-pair approach gave a noticeable increase in 3-D similarity scores, compared to the single-conformer approach. However, the magnitude of this increase was comparable to those for the random compound pairs, meaning that employing multiple conformers per compound does not increase the separation between the NN-pairs and random compound pairs on average. On the basis of these observations, it was inferred that the multiple-conformer approach would not also result in a noticeable separation between the NI- and NN-pairs “on average”.

The present study is a critical step to understand effects of conformational diversity of the molecules upon the 3-D molecular similarity and its application to aggregated biological assay data analysis. The results of this study may be helpful to build search and analysis tools that exploit 3-D molecular similarity between compounds archived in PubChem and other molecular libraries in a statistically meaningful way.

## Methods

### Datasets

The previous study [10] employed the 734-K set, consisting of 734,485 biologically tested compounds with 3-D information available, to investigate the distributions of the 3-D similarity scores between randomly selected compounds, using a single conformer per compound. However, the 734-K set is so large that it is not currently practical to employ multiple conformers for each molecule. Therefore, the 10-K set (Additional file 1) was constructed by randomly selecting 10,000 compounds from the 734-K set. As shown in Table 1, the 10-K set and the 734-K set have nearly identical average values and standard deviations for various molecular properties, such as the molecular volumes, steric shape quadrupole moments, heavy atom count, and feature counts, and are comparable to the entire PubChem3D contents.

The 156-K set consists of 156,232 CIDs (Additional file 2) that have 3-D information available *and* that are declared as “non-inactives” in any assays archived in the PubChem BioAssay database as of late January 2010 (ranging from AID 1 to AID 2310). [A non-inactive molecule is defined as any molecule that is not inactive against the assay target, including “unspecified/inconclusive” compounds as well as “active” compounds]. This set was used to investigate the distribution of the 3-D similarity scores between the NN pairs for a given assay. For these per-assay analyses, it is desirable to exclude bioassays with too small number of non-inactives, because those small-size assays may cause biased results. However, note that the non-inactive count of a bioassay depends upon the assay type. For example, while primary (high-throughput) screenings typically may have up to thousands of non-inactives, summary assays may have only a few non-inactives. Because summary assays, which correspond to the last stage of probe/lead discovery, are typically more accurate than other assays, we wanted to include as many of them in our analysis as possible. Therefore, it was necessary to determine an appropriate non-inactive count threshold (or a NN-pair count threshold) to exclude small-size assays without losing too many summary assays. In our previous study [10], bioassays with less than six NN pairs or less than NI pairs were excluded, minimizing the loss of summary assays. Similarly, the present study considered 1,528 bioassays with at least six NN pairs (Figure 8).

Note that the non-inactive compounds of these 1,528 bioassays include the inconclusive and unspecified compounds as well as the active compounds. The reason for using the non-inactive compounds instead of the active compounds in our analysis is that the inconclusive and unspecified compounds are indeed active in many assays. The use of the non-inactive compounds somehow reflects the heterogeneous nature of the PubChem Bioassay data, because the activity outcome of the compounds tested in PubChem bioassays is determined by the individual depositors. This choice may raise a concern especially when one is interested in a separation of the “nominal” active space from the inactive space. Therefore, a subset of the 1,528 assays were generated by selecting 843 assays which have active compounds only (without any inconclusive or unspecified compounds), and the same analyses were performed both for the 1,528 assay set and the 843 assay set. The results from the 843 assay set are included in Additional file 4. The similarity score distributions from the two assay sets have very similar averages and standard deviations to each other, and hence, the conclusions drawn from the 1,528 assay set should also be valid for the 843 assay set.

### Conformer models

The computed 3-D conformer models for compounds in the 10-K and 156-K sets were downloaded from PubChem. The PubChem3D conformer generation and sampling procedures are explained in detail in our recent studies [6,11,56] and only a brief description is given below. The PubChem3D conformer models are generated using the OMEGA software from the OpenEye Scientific Software, Inc. In an initial stage, a maximum of 100,000 conformers are generated for each chemical structure, using the Merck Molecular Force Field (MMFF94s) minus the coulombic terms, and an energy filter of 25 kcal/mol. If the chemical structure has undefined stereo centers, a maximum of 100,000 conformers are generated for each of the stereo isomers arising from enumeration of the undefined stereo centers, and then combined together. [Therefore, a molecule with five undefined stereo centers will have 32 (= 2^5^) stereo isomers, resulting a maximum of 3.2 million conformers being considered].

Because it is not practical to store all the conformers and to use them efficiently, the conformer models are down-sampled through conformer clustering with an RMSD clustering threshold (*RMSD*^*thresh*^) determined by the following equations:

(1) $$RMSD^{\mathrm{thresh}}=\frac{int\left(0.5+RMSD^{\mathrm{pred}}\times 5\right)}{5}$$

(2) $$RMSD^{\mathrm{pred}}=0.219+0.0099\times N_{\mathrm{NHA}}+0.040\times N_{\mathrm{ER}}$$

(3) $$N_{\mathrm{ER}}=N_{R}+\frac{N_{\mathrm{NARA}}}{5}$$

where *N*_*NHA*_ is the number of non-hydrogen (heavy) atoms, *N*_*R*_ is the number of rotatable bonds, *N*_*NARA*_ is the number of non-aromatic *sp*^3^-hybridized ring atoms, and *N*_*ER*_ is the effective rotor count of a molecule, which takes into account molecular flexibility due to rotatable bonds and ring flexibility simultaneously. *RMSD*^*pred*^ is the predicted upper limit of the conformer model accuracy to ensure at least 90% of the PubChem3D conformer models have at least one “bioactive” conformer whose RMSD distance from the experimentally determined conformation is closer than *RMSD*^*pred*^. If the conformer sampling with *RMSD*^*thresh*^, which is the *RMSD*^*pred*^ value rounded to the nearest 0.2 increment [Equation (1)], results in more than 500 conformers for a molecule, the *RMSD*^*thresh*^ value is incremented by a further 0.2 and the conformer model is re-clustered. This process is repeated as many times as necessary to restrict the overall count of conformers to be 500 or less. This conformer sampling stage reduces the size of the conformer models without significant loss of the conformer model accuracy [56].

After conformer model sampling, a post processing step is performed to completely relax the hydrogen atom locations by performing an energy minimization with all non-hydrogen atoms frozen. A subsequent "bump" check removes any conformers that have MMFF94 atom-atom interactions greater than 25 kcal/mol. Each conformer is rotated and translated to their principal steric axes (*i.e*., non-mass weighted principal moments of inertia axes), considering only non-hydrogen atoms. A diverse conformer ordering gives a maximal description of the conformational space spanned by a molecule, if only a subset of conformers are used.

Note that the conformers produced are not stationary points on a potential energy hypersurface. Instead, the PubChem3D conformer model for a chemical structure is meant to represent all possible biologically-relevant conformations that the molecule may have. In theory, one should have a reasonable chance (~90%) to find any biologically accessible conformation within the RMSD sampling distance of the conformer model [6,56].

Although all of the (up to 500) conformers for each molecule are accessible from the PubChem website (http://pubchem.ncbi.nlm.nih.gov), only the first ten “diverse” conformers per compound [11] are available for public bulk download via the PubChem FTP site (ftp://pubchem.ncbi.nlm.nih.gov). In addition, most search and analysis tools provided by PubChem3D use up to ten conformers per compound. Therefore, the present study only considered the first ten diverse conformers for similarity score computation.

### ROCS-based similarity scores used in PubChem3D

Among many 3-D structure comparison approaches, PubChem uses ROCS [14,21] from the OpenEye Scientific Software, Inc. Because ROCS uses atom-centered Gaussian functions to describe the molecular shape [19,20], it can perform a rapid shape superposition without a considerable loss of accuracy, compared to when the hard-sphere volumes is employed. In recent studies [15,57], ROCS was shown to be comparable with, and often better than, structure-based approaches in virtual screening, both in terms of overall performance and consistency [17].

To quantify 3-D similarity between molecules, two 3-D similarity measures are used: shape-Tanimoto (ST) [8,10,11,14,19-21] and color-Tanimoto (CT) [8,10,11,19,20]. The ST score is a measure of shape similarity, which is defined as the following:

(4) $$ST=\frac{V_{\mathrm{AB}}}{V_{\mathrm{AA}}+V_{\mathrm{BB}}-V_{\mathrm{AB}}}$$

where *V*_*AA*_ and *V*_*BB*_ are the respective self-overlap volumes of conformers A and B and *V*_*AB*_ is the overlap volume between conformers A and B. The CT score, given by Equation (5), quantifies the similarity of 3-D orientation of protein-binding “features” between conformers, by checking the overlap of “fictitious” feature atoms (also called “color” atoms) used to represent the six types of functional groups considered: hydrogen-bond donors, hydrogen-bond acceptors, cations, anions, hydrophobes, and rings [19,20].

(5) $$CT=\frac{\sum_{f}V_{\mathrm{AB}}^{f}}{\sum_{f}V_{\mathrm{AA}}^{f}+\sum_{f}V_{\mathrm{BB}}^{f}-\sum_{f}V_{\mathrm{AB}}^{f}}$$

where: the index “*f*” indicates any of the six independent fictitious feature atom types; *V*_*AA*_^*f*^ and *V*_*BB*_^*f*^ are the self-overlap volumes of conformers A and B for feature atom type *f*, respectively; and *V*_*AB*_^*f*^ is the overlap volume between conformers A and B for feature atom type *f*. Additionally, to consider shape similarity and feature similarity simultaneously, the two similarity metrics can be combined to create a so-called combo-Tanimoto (ComboT) [10,11,19,20], as specified by Equation (6):

(6) ComboT=ST+CT

Because the ST and CT scores range from 0 (for no similarity) to 1 (for identifical molecules) by definition, the ComboT score ranges from 0 to 2 (without normalization to unity, due to pre-existing convention). Two different conformer superpositions are used: (1) the ST-optimized (or shape-optimized) superposition, where the ST score between conformers is maximized, and (2) the CT-optimized (or feature-optimized) superposition, where the CT score between conformers is maximized. As a result, PubChem3D quantifies 3-D molecular similarity using six different scores: *ST*^*ST-opt*^, *CT*^*ST-opt*^, *ComboT*^*ST-opt*^, *ST*^*CT-opt*^, *CT*^*CT-opt*^, and *ComboT*^*CT-opt*^, where superscripts “ST-opt” and “CT-opt” indicate the ST-optimization and CT-optimization, respectively.

### 3-D similarity score computation

The six 3-D similarity measures (*i.e.*, *ST*^*ST-opt*^, *CT*^*ST-opt*^, *ComboT*^*ST-opt*^, *ST*^*CT-opt*^, *CT*^*CT-opt*^, and *ComboT*^*CT-opt*^) were computed using the C++ Shape toolkit [20]. For the 10-K set, a full (all-by-all) similarity matrix was computed for each of the six similarity measures. However, it was not practical to compute a full similarity score matrix for the 156-K set using multiple conformers per compound. Therefore, the 3-D similarity scores between two compounds in the 156-K set were computed only when both molecules were tested as non-inactives in at least one common bioassay. The computed similarity scores for each conformer pair were stored with the translation/rotation matrix that yields the corresponding alignment. The computed similarity scores were extracted from the data files, and histograms of the similarity scores were generated after binning all similarity scores to their nearest 0.01 increment.

In the second part of this study, the average and standard deviation of the 3-D similarity scores between the NN-pairs were computed for each of the 1,528 assays. These per-AID average and standard deviation are denoted with Greek letters *μ* and *σ*, respectively, followed by the corresponding similarity measure in parentheses [*e.g.*, *μ*(*ComboT*_*single*_^*ST-opt*^) and *σ*(*ComboT*_*single*_^*ST-opt*^)]. The distributions of the *μ*(*XT*) values for the 1,528 assays considered in this study are shown in Figures 9 and 10, for the ST-optimized and CT-optimized 3-D similarities, respectively.

To investigate the multiple-conformer effect upon the per-AID NN-pair similarity scores, it was necessary to compare the 3-D similarity score distributions from the single-conformer approach with those from the multiple-conformer approach. Therefore, the average and standard deviation of the similarity score difference between two conformer models for a given AID were computed using the following equations:

(7) $$\mu \left(XT_{multi-single}\right)=\mu \left(XT_{\mathrm{multi}}\right)-\mu \left(XT_{\mathrm{single}}\right)$$

(8) $$\sigma \left(XT_{multi-single}\right)=\sqrt{\frac{{\left[\sigma \left(XT_{\mathrm{multi}}\right)\right]}^{2}}{n_{\mathrm{multi}}}+\frac{{\left[\sigma \left(XT_{\mathrm{single}}\right)\right]}^{2}}{n_{\mathrm{single}}}}$$

where “single” and “multi” indicate the type of conformer models (*i.e.*, the single-conformer or multiple-conformer approach), and *n*_*single*_ and *n*_*multi*_ are the number of the compound pairs used in the single- and multiple-conformer approaches, respectively, for a given assay. Because two different approaches (the best-conformer-pair and all-conformer-pair approaches) were employed for the multiple-conformer approach, subscripts “best” and “all” are also used to distinguish them for clarification. Note that *n*_*single*_ and *n*_*multi*_ are not necessarily the same as each other for a given assay, because the similarity score distributions for the all-conformer-pair approach considers as up to 100 times many conformer pairs as the single-conformer approach.

When we refer to the average and standard deviation of a set of the per-AID statistical parameters (*e.g.,* over all the 1,528 AIDs), we use additional Greek letters *μ* and *σ*, respectively, followed by the corresponding statistical parameters in brackets. For example, *μ*[*μ*(*ST*_*best*−*single*_^*ST-opt*^)] and *σ*[*μ*(*ST*_*best*−*single*_^*ST-opt*^)] indicate the overall average and standard deviation of *μ*(*ST*_*best*−*single*_^*ST-opt*^) over a set of AIDs. Note that the standard deviation for the *μ*(*XT*) and *σ*(*XT*) values (*i.e., σ*[*μ*(*XT*)] and *σ*[*σ*(*XT*)]) were computed using the following equations:

(9) $$\sigma \left[\mu \left(XT\right)\right]=\sqrt{\frac{\sum_{i}^{N}{\left(\mu_{i}\left(XT\right)-\mu \left[\mu \left(XT\right)\right]\right)}^{2}}{N-1}}$$

(10) $$\sigma \left[\sigma \left(XT\right)\right]=\sqrt{\frac{\sum_{i}^{N}{\left(\sigma_{i}\left(XT\right)-\mu \left[\sigma \left(XT\right)\right]\right)}^{2}}{N-1}}$$

where *μ*_*i*_(*XT*) and *σ*_*i*_(*XT*) are the average and standard deviation of the NN-pair similarity scores for an assay *i* and *N* is the number of assays considered.

## Competing interests

The authors declare that they have no competing interests.

## Authors’ contributions

EEB computed the similarity score matrices. SK analyzed the data and wrote the first draft. SHB reviewed the final manuscript. All authors read and approved the final manuscript.

## Supplementary Material

Additional file 1 **10-K CID set.** A list of 10,000 CIDs, used for construction of the 3-D similarity score distributions for the random compound pairs. Click here for file

Additional file 2 **156-K CID set.** A list of 156,232 CIDs, used to investigate the NN-pair 3-D similarity scores on the per-assay basis. Click here for file

Additional file 3 **Similarity Scores.** A tab-delimited file that contains statistical parameters of the NN-pair 3-D similarity scores for each of 1,528 AIDs considered for per-assay analysis. Click here for file

Additional file 4 **Analysis of the 843 assays.** Supplementary figures and tables that summarize the results from the 843 assays that have active compounds only (without any inconclusive or unspecified compounds). Click here for file

Additional file 5 **Conversion map from the 3-D similarity score to the p-value.** An excel file that contains the conversion maps from a 3-D similarity score between molecules to the *p*-value of getting that score by chance. Click here for file
