# Supplementary material for: Effects of multiple conformers per compound upon 3-D similarity search and bioassay data analysis
Source: J Cheminform. 2012 Nov 7;4:28. doi: 10.1186/1758-2946-4-28 (PMC3537644; doi:10.1186/1758-2946-4-28)
Supplement: Additional file 4 — Analysis of the 843 assays. Supplementary figures and tables that summarize the results from the 843 assays that have active compounds only (without any inconclusive or unspecified compounds). [file 1758-2946-4-28-S4.docx]

**
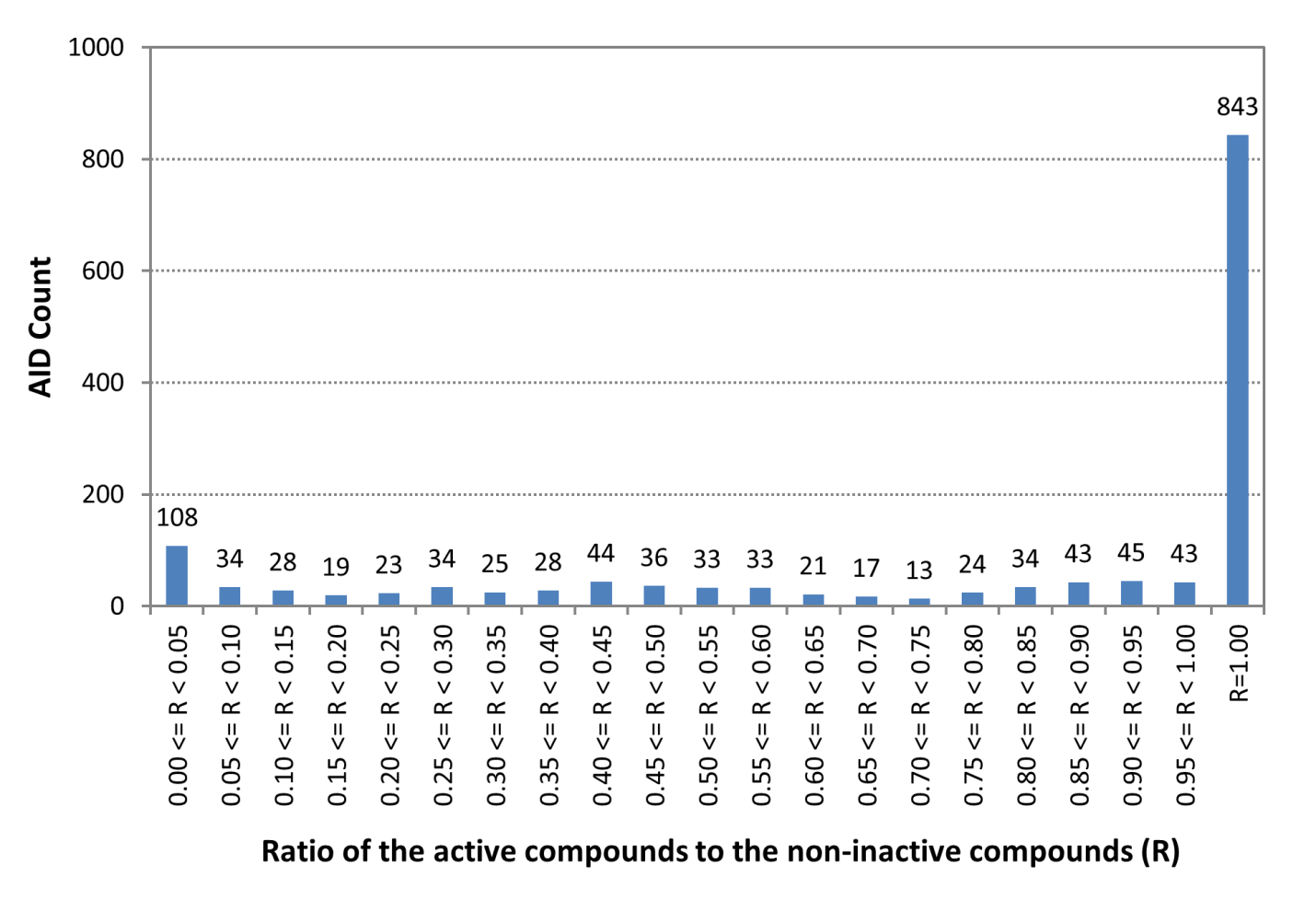
**

**Figure S1. Frequencies of the ratio (R) of the active compounds to the non-inactive compounds.** Among 1,528 biological assays considered in the present study, 843 assays have active compounds only (with no inconclusive or unspecified compounds), whereas 108 assays have no active compounds.

**
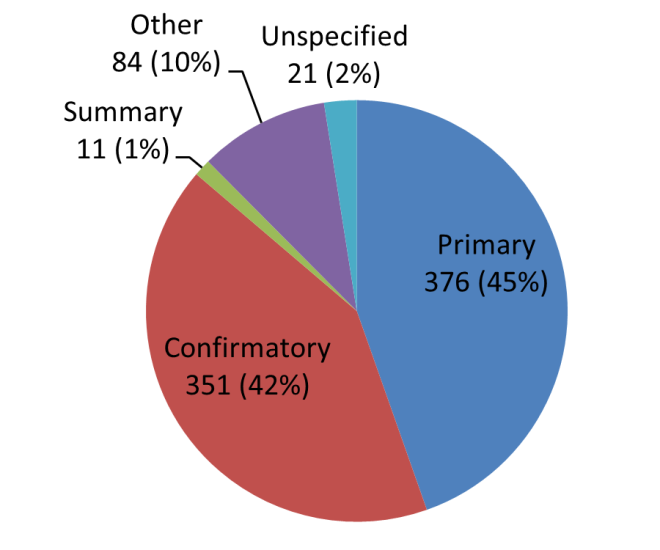
**

**Figure S2**. **Break down of the 843 assays by type.** Assay-type counts for the 843 bioassays that have active compounds only (with no inconclusive or unspecified compounds).


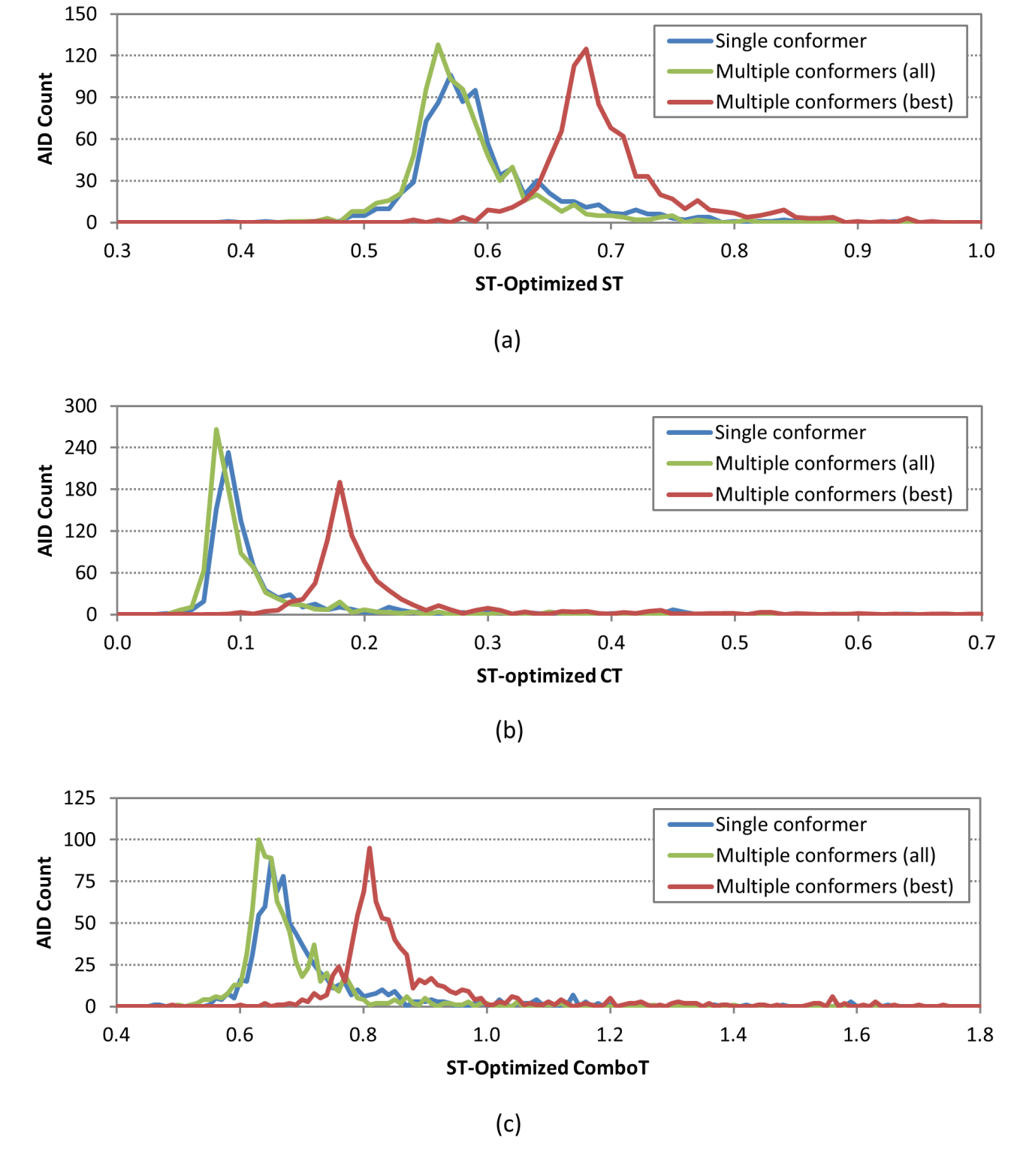


**Figure S3. Per-AID shape-Tanimoto (ST) optimized average values for the 843 AIDs.** Binned distributions in 0.01 increments of the average optimized similarity scores for non-inactive–non-inactive (NN) pairs of 843 AIDs in the PubChem BioAssay database, computed at the shape-Tanimoto-optimized superposition: (a) shape-Tanimoto (ST), (b) color-Tanimoto (CT), and (c) combo-Tanimoto (ComboT). “Single conformer”, “Multiple-conformers (all)”, and “Multiple conformers (best)” correspond to search scenarios *A*, *B*, and *E*, respectively (See **Table 1**).


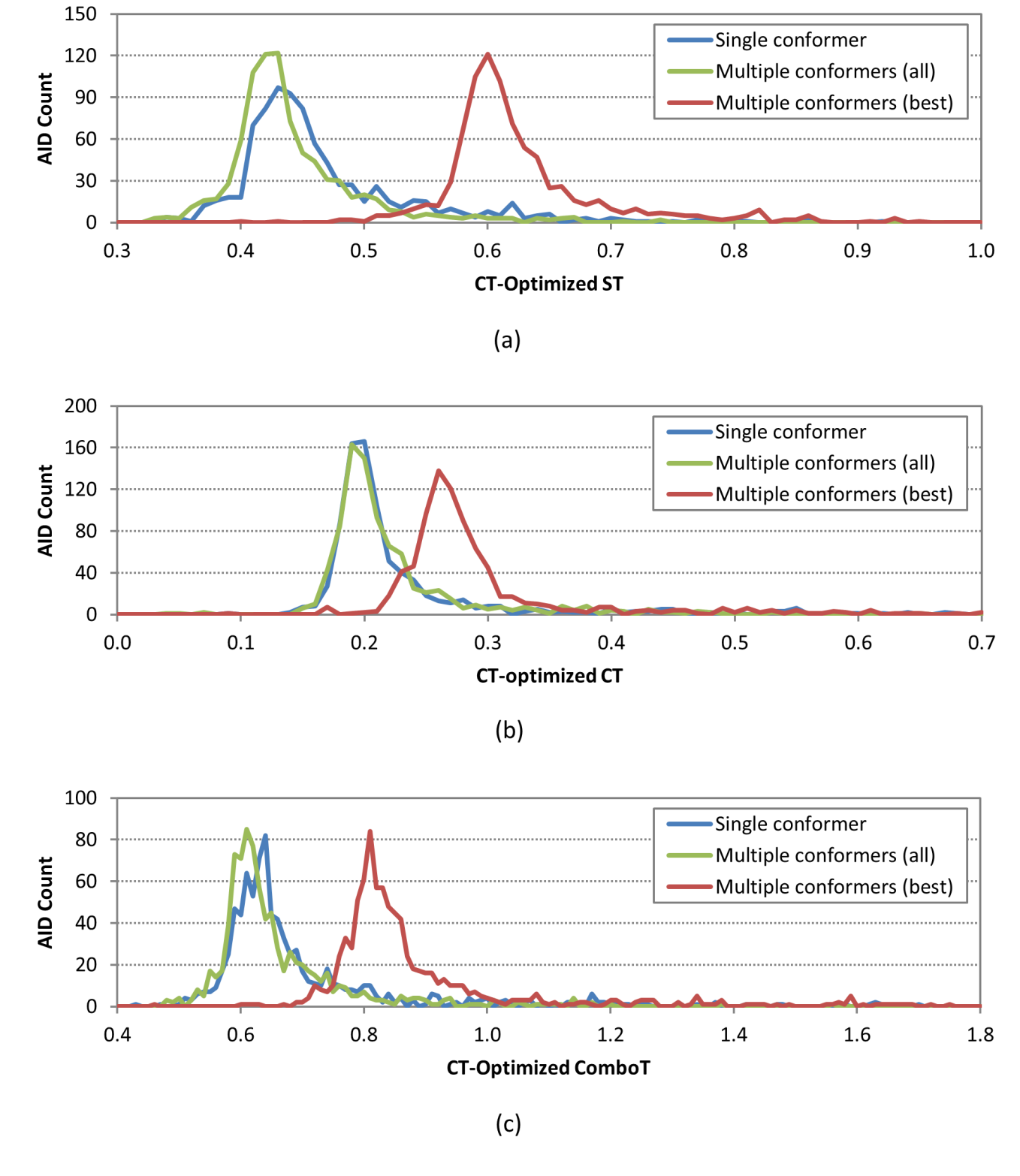


**Figure S4. Per-AID color-Tanimoto (CT) optimized average values for the 843 AIDs.** Binned distributions in 0.01 increments of the average optimized similarity scores for non-inactive–non-inactive (NN) pairs of 843 AIDs in the PubChem BioAssay database, computed at color-Tanimoto-optimized superposition: (a) shape-Tanimoto (ST), (b) color-Tanimoto (CT), and (c) combo-Tanimoto (ComboT). “Single conformer”, “Multiple-conformers (all)”, and “Multiple conformers (best)” correspond to search scenarios *A*, *B*, and *E*, respectively (See **Table 1**).


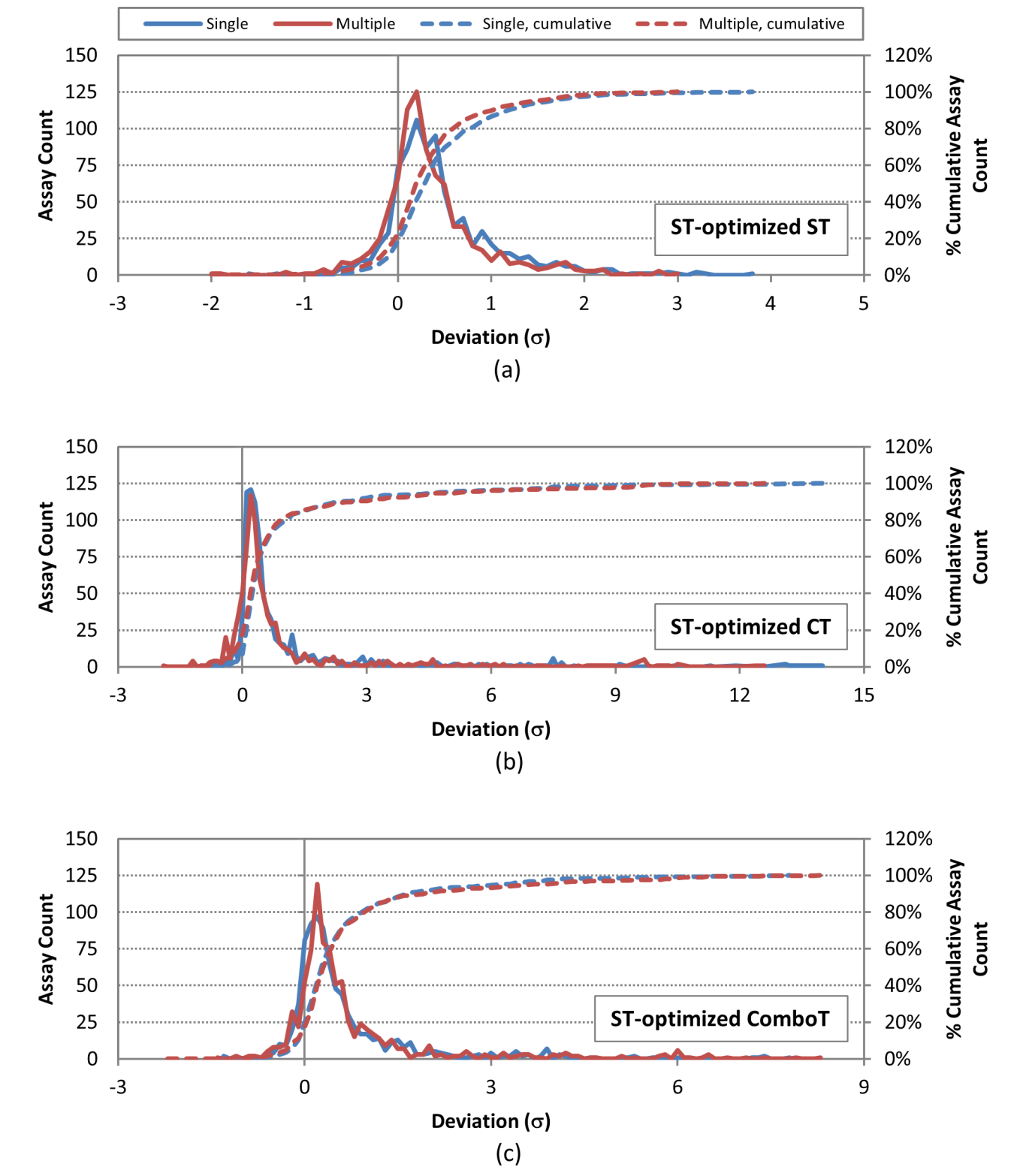


**Figure 11**. **Deviation from random of per-AID shape-Tanimoto (ST) optimized average values for the 843 AIDs.** Deviation of the ST-optimized similarity scores for non-inactive–non-inactive (NN) pairs of 843 AIDs from the corresponding average for the random compound pairs, computed using both a single conformer and best multiple (ten) diverse conformers per compound: (a) ST-optimized ST, (b) ST-optimized CT, and (c) ST-optimized ComboT. The deviations are binned with increment of 0.1 standard deviation (σ) unit. “Single” and “Multiple” refer to search scenarios *A* and *E*, respectively (See **Table 1**).


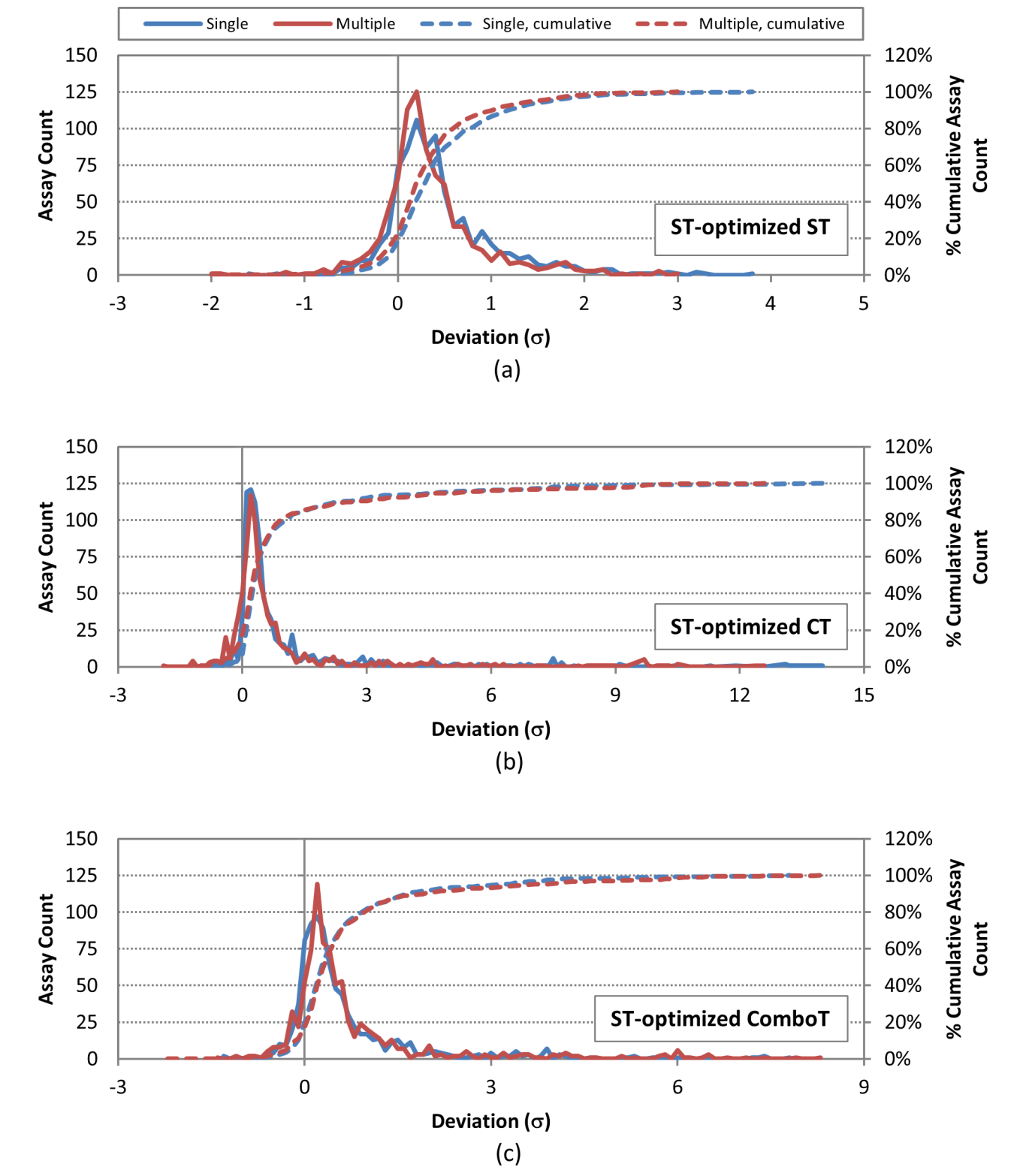


**Figure 12**. **Deviation from random of per-AID color-Tanimoto (CT) optimized average values for the 843 AIDs.** Deviation of the CT-optimized similarity scores for non-inactive-non-inactive (NN) pairs of 843 AIDs from the corresponding average for the random compound pairs, computed using both a single conformer and best multiple (ten) diverse conformers per compound: (a) CT-optimized ST, (b) CT-optimized CT, and (c) CT-optimized ComboT. The deviations are binned with increment of 0.1 standard deviation (σ) unit. “Single” and “Multiple” refer to search scenarios *A* and *E*, respectively (See **Table 1**).

**Table S1. Summary statistics for per-AID shape-Tanimoto (ST) optimized 3-D similarity for the 843 bioassays.**

| Assay | ST*^ST-opt^* | | |  | CT*^ST-opt^* | | |  | ComboT*^ST-opt^* | | |
| --- | --- | --- | --- | --- | --- | --- | --- | --- | --- | --- | --- |
| Type | Single | All | Best |  | Single | All | Best |  | Single | All | Best |
| **μ[μ(XT)]** |  |  |  |  |  |  |  |  |  |  |  |
| Screening | 0.58 | 0.57 | 0.69 |  | 0.10 | 0.09 | 0.20 |  | 0.69 | 0.66 | 0.85 |
| Confirmatory | 0.60 | 0.58 | 0.70 |  | 0.14 | 0.11 | 0.24 |  | 0.74 | 0.69 | 0.90 |
| Summary | 0.62 | 0.61 | 0.72 |  | 0.22 | 0.19 | 0.33 |  | 0.85 | 0.80 | 1.01 |
| Other | 0.58 | 0.57 | 0.68 |  | 0.12 | 0.09 | 0.24 |  | 0.70 | 0.66 | 0.89 |
| Unspecified | 0.58 | 0.56 | 0.65 |  | 0.10 | 0.08 | 0.16 |  | 0.67 | 0.64 | 0.78 |
| All assays | 0.59 | 0.58 | 0.69 |  | 0.12 | 0.10 | 0.22 |  | 0.71 | 0.67 | 0.87 |
| *Random* | *0.54* | *0.54* | *0.65* |  | *0.07* | *0.07* | *0.16* |  | *0.62* | *0.61* | *0.77* |
|  |  |  |  |  |  |  |  |  |  |  |  |
| **σ[μ(XT)]** |  |  |  |  |  |  |  |  |  |  |  |
| Screening | 0.04 | 0.03 | 0.04 |  | 0.06 | 0.03 | 0.10 |  | 0.10 | 0.06 | 0.13 |
| Confirmatory | 0.07 | 0.05 | 0.06 |  | 0.11 | 0.05 | 0.13 |  | 0.17 | 0.10 | 0.18 |
| Summary | 0.11 | 0.09 | 0.08 |  | 0.21 | 0.17 | 0.22 |  | 0.31 | 0.26 | 0.31 |
| Other | 0.07 | 0.06 | 0.08 |  | 0.08 | 0.05 | 0.14 |  | 0.15 | 0.10 | 0.22 |
| Unspecified | 0.06 | 0.04 | 0.05 |  | 0.04 | 0.04 | 0.04 |  | 0.09 | 0.08 | 0.08 |
| All assays | 0.06 | 0.05 | 0.06 |  | 0.09 | 0.05 | 0.12 |  | 0.14 | 0.09 | 0.17 |
|  |  |  |  |  |  |  |  |  |  |  |  |
| **μ[σ(XT)]** |  |  |  |  |  |  |  |  |  |  |  |
| Screening | 0.10 | 0.09 | 0.09 |  | 0.08 | 0.07 | 0.08 |  | 0.16 | 0.14 | 0.15 |
| Confirmatory | 0.10 | 0.09 | 0.09 |  | 0.12 | 0.10 | 0.12 |  | 0.20 | 0.17 | 0.19 |
| Summary | 0.10 | 0.09 | 0.09 |  | 0.14 | 0.13 | 0.14 |  | 0.22 | 0.20 | 0.21 |
| Other | 0.11 | 0.09 | 0.10 |  | 0.12 | 0.09 | 0.12 |  | 0.21 | 0.16 | 0.21 |
| Unspecified | 0.13 | 0.12 | 0.13 |  | 0.09 | 0.08 | 0.10 |  | 0.19 | 0.17 | 0.20 |
| All assays | 0.10 | 0.09 | 0.09 |  | 0.10 | 0.08 | 0.10 |  | 0.18 | 0.15 | 0.17 |
| *Random* | *0.10* | *0.09* | *0.10* |  | *0.05* | *0.05* | *0.06* |  | *0.13* | *0.12* | *0.13* |
|  |  |  |  |  |  |  |  |  |  |  |  |
| **σ[σ(XT)]** |  |  |  |  |  |  |  |  |  |  |  |
| Screening | 0.02 | 0.01 | 0.01 |  | 0.05 | 0.03 | 0.04 |  | 0.07 | 0.04 | 0.05 |
| Confirmatory | 0.02 | 0.01 | 0.02 |  | 0.07 | 0.05 | 0.07 |  | 0.09 | 0.05 | 0.09 |
| Summary | 0.02 | 0.01 | 0.02 |  | 0.08 | 0.07 | 0.08 |  | 0.09 | 0.08 | 0.10 |
| Other | 0.03 | 0.02 | 0.03 |  | 0.07 | 0.04 | 0.06 |  | 0.09 | 0.05 | 0.08 |
| Unspecified | 0.02 | 0.03 | 0.03 |  | 0.05 | 0.05 | 0.06 |  | 0.07 | 0.07 | 0.09 |
| All assays | 0.02 | 0.01 | 0.02 |  | 0.07 | 0.04 | 0.06 |  | 0.08 | 0.05 | 0.08 |

The overall average and standard deviation of the AID-specific average and standard deviation as a function of search scenario and per assay type classifier. “All assays” corresponds to all assays irrespective of assay type. “*Random*” corresponds to the 10-K set results found from **Table 3** as a means of comparison. “Single”, “All”, and “Best” correspond to search scenarios “A”, “B”, and “E” in **Table 1**, respectively.

**Table S2. Summary statistics for per-AID color-Tanimoto (CT) optimized 3-D similarity for the 843 bioassays.**

| Assay | ST*^CT-opt^* | | |  | CT*^CT-opt^* | | |  | ComboT*^CT-opt^* | | |
| --- | --- | --- | --- | --- | --- | --- | --- | --- | --- | --- | --- |
| Type | Single | All | Best |  | Single | All | Best |  | Single | All | Best |
| **μ[μ(XT)]** |  |  |  |  |  |  |  |  |  |  |  |
| Screening | 0.45 | 0.43 | 0.61 |  | 0.21 | 0.20 | 0.28 |  | 0.66 | 0.63 | 0.85 |
| Confirmatory | 0.48 | 0.45 | 0.63 |  | 0.24 | 0.23 | 0.32 |  | 0.72 | 0.68 | 0.91 |
| Summary | 0.51 | 0.49 | 0.66 |  | 0.31 | 0.32 | 0.40 |  | 0.82 | 0.81 | 1.02 |
| Other | 0.46 | 0.43 | 0.61 |  | 0.24 | 0.22 | 0.32 |  | 0.70 | 0.65 | 0.90 |
| Unspecified | 0.47 | 0.44 | 0.58 |  | 0.20 | 0.19 | 0.24 |  | 0.66 | 0.62 | 0.78 |
| All assays | 0.46 | 0.44 | 0.62 |  | 0.23 | 0.22 | 0.30 |  | 0.69 | 0.65 | 0.88 |
| *Random* | *0.41* | *0.40* | *0.57* |  | *0.18* | *0.18* | *0.25* |  | *0.59* | *0.58* | *0.77* |
|  |  |  |  |  |  |  |  |  |  |  |  |
| **σ[μ(XT)]** |  |  |  |  |  |  |  |  |  |  |  |
| Screening | 0.05 | 0.04 | 0.05 |  | 0.06 | 0.04 | 0.09 |  | 0.11 | 0.07 | 0.14 |
| Confirmatory | 0.09 | 0.06 | 0.07 |  | 0.11 | 0.07 | 0.12 |  | 0.20 | 0.12 | 0.19 |
| Summary | 0.15 | 0.13 | 0.10 |  | 0.22 | 0.21 | 0.21 |  | 0.36 | 0.33 | 0.33 |
| Other | 0.09 | 0.07 | 0.09 |  | 0.10 | 0.07 | 0.14 |  | 0.17 | 0.13 | 0.22 |
| Unspecified | 0.06 | 0.04 | 0.05 |  | 0.05 | 0.05 | 0.04 |  | 0.10 | 0.09 | 0.09 |
| All assays | 0.08 | 0.05 | 0.07 |  | 0.09 | 0.06 | 0.11 |  | 0.16 | 0.11 | 0.18 |
|  |  |  |  |  |  |  |  |  |  |  |  |
| **μ[σ(XT)]** |  |  |  |  |  |  |  |  |  |  |  |
| Screening | 0.13 | 0.11 | 0.10 |  | 0.08 | 0.08 | 0.09 |  | 0.18 | 0.16 | 0.16 |
| Confirmatory | 0.13 | 0.12 | 0.10 |  | 0.11 | 0.11 | 0.12 |  | 0.22 | 0.20 | 0.20 |
| Summary | 0.13 | 0.12 | 0.10 |  | 0.12 | 0.12 | 0.13 |  | 0.23 | 0.22 | 0.22 |
| Other | 0.14 | 0.12 | 0.11 |  | 0.12 | 0.10 | 0.12 |  | 0.23 | 0.19 | 0.21 |
| Unspecified | 0.14 | 0.13 | 0.13 |  | 0.10 | 0.10 | 0.11 |  | 0.21 | 0.19 | 0.21 |
| All assays | 0.13 | 0.12 | 0.10 |  | 0.10 | 0.09 | 0.10 |  | 0.20 | 0.18 | 0.18 |
| *Random* | *0.11* | *0.11* | *0.10* |  | *0.06* | *0.06* | *0.07* |  | *0.14* | *0.13* | *0.14* |
|  |  |  |  |  |  |  |  |  |  |  |  |
| **σ[σ(XT)]** |  |  |  |  |  |  |  |  |  |  |  |
| Screening | 0.02 | 0.01 | 0.02 |  | 0.04 | 0.03 | 0.03 |  | 0.07 | 0.04 | 0.05 |
| Confirmatory | 0.03 | 0.02 | 0.03 |  | 0.06 | 0.04 | 0.06 |  | 0.08 | 0.06 | 0.09 |
| Summary | 0.02 | 0.01 | 0.02 |  | 0.07 | 0.05 | 0.08 |  | 0.09 | 0.06 | 0.11 |
| Other | 0.03 | 0.02 | 0.03 |  | 0.05 | 0.04 | 0.05 |  | 0.08 | 0.06 | 0.08 |
| Unspecified | 0.02 | 0.03 | 0.03 |  | 0.05 | 0.05 | 0.05 |  | 0.07 | 0.08 | 0.09 |
| All assays | 0.03 | 0.02 | 0.02 |  | 0.05 | 0.04 | 0.05 |  | 0.08 | 0.05 | 0.08 |

The overall average and standard deviation of the AID-specific average and standard deviation as a function of search scenario and per assay type classifier. “All assays” corresponds to all assays irrespective of assay type. “*Random*” corresponds to the 10-K set results found from **Table 3** as a means of comparison. “Single”, “All”, and “Best” correspond to search scenarios “A”, “B”, and “E” in **Table 1**, respectively.

**Table S3. Comparison of summary statistics for per-AID 3-D similarity for the 843 bioassays.**

|  | ST-optimized | | | | | | |  | CT-optimized | | | | | | |
| --- | --- | --- | --- | --- | --- | --- | --- | --- | --- | --- | --- | --- | --- | --- | --- |
| Assay | Best − Single | | |  | All − Single | | |  | Best − Single | | |  | All − Single | | |
| Type | ST | CT | ComboT |  | ST | CT | ComboT |  | ST | CT | ComboT |  | ST | CT | ComboT |
| **μ[μ(XT)]** |  |  |  |  |  |  |  |  |  |  |  |  |  |  |  |
| Primary | 0.10 | 0.09 | 0.16 |  | -0.01 | -0.01 | -0.03 |  | 0.16 | 0.07 | 0.19 |  | -0.02 | 0.00 | -0.02 |
| Confirmatory | 0.09 | 0.10 | 0.16 |  | -0.02 | -0.03 | -0.05 |  | 0.15 | 0.07 | 0.18 |  | -0.03 | -0.01 | -0.04 |
| Summary | 0.09 | 0.10 | 0.16 |  | -0.03 | -0.05 | -0.08 |  | 0.14 | 0.08 | 0.19 |  | -0.04 | -0.01 | -0.04 |
| Other | 0.09 | 0.11 | 0.16 |  | -0.01 | -0.02 | -0.03 |  | 0.15 | 0.07 | 0.18 |  | -0.02 | -0.02 | -0.04 |
| Unspecified | 0.06 | 0.06 | 0.10 |  | 0.00 | -0.01 | -0.01 |  | 0.12 | 0.04 | 0.13 |  | -0.02 | 0.00 | -0.01 |
| All assays | 0.09 | 0.09 | 0.15 |  | -0.01 | -0.02 | -0.04 |  | 0.15 | 0.07 | 0.18 |  | -0.02 | -0.01 | -0.03 |
|  |  |  |  |  |  |  |  |  |  |  |  |  |  |  |  |
| **σ[μ(XT)]** |  |  |  |  |  |  |  |  |  |  |  |  |  |  |  |
| Primary | 0.02 | 0.04 | 0.05 |  | 0.02 | 0.04 | 0.05 |  | 0.03 | 0.03 | 0.05 |  | 0.03 | 0.03 | 0.05 |
| Confirmatory | 0.03 | 0.04 | 0.06 |  | 0.04 | 0.08 | 0.12 |  | 0.04 | 0.04 | 0.06 |  | 0.06 | 0.06 | 0.12 |
| Summary | 0.04 | 0.04 | 0.06 |  | 0.03 | 0.07 | 0.10 |  | 0.05 | 0.03 | 0.07 |  | 0.04 | 0.04 | 0.07 |
| Other | 0.03 | 0.07 | 0.09 |  | 0.02 | 0.04 | 0.06 |  | 0.04 | 0.05 | 0.08 |  | 0.03 | 0.04 | 0.07 |
| Unspecified | 0.01 | 0.01 | 0.02 |  | 0.02 | 0.01 | 0.02 |  | 0.02 | 0.01 | 0.02 |  | 0.02 | 0.01 | 0.03 |
| All assays | 0.03 | 0.04 | 0.06 |  | 0.03 | 0.06 | 0.09 |  | 0.04 | 0.04 | 0.06 |  | 0.04 | 0.05 | 0.09 |
|  |  |  |  |  |  |  |  |  |  |  |  |  |  |  |  |
| **μ[σ(XT)]** |  |  |  |  |  |  |  |  |  |  |  |  |  |  |  |
| Primary | 0.01 | 0.01 | 0.01 |  | 0.00 | 0.01 | 0.01 |  | 0.01 | 0.01 | 0.01 |  | 0.01 | 0.00 | 0.01 |
| Confirmatory | 0.01 | 0.02 | 0.02 |  | 0.01 | 0.01 | 0.02 |  | 0.01 | 0.01 | 0.02 |  | 0.01 | 0.01 | 0.02 |
| Summary | 0.02 | 0.03 | 0.05 |  | 0.02 | 0.02 | 0.04 |  | 0.02 | 0.03 | 0.05 |  | 0.02 | 0.02 | 0.04 |
| Other | 0.01 | 0.01 | 0.01 |  | 0.00 | 0.01 | 0.01 |  | 0.01 | 0.01 | 0.02 |  | 0.01 | 0.01 | 0.01 |
| Unspecified | 0.01 | 0.01 | 0.01 |  | 0.01 | 0.00 | 0.01 |  | 0.01 | 0.01 | 0.01 |  | 0.01 | 0.00 | 0.01 |
| All assays | 0.01 | 0.01 | 0.02 |  | 0.01 | 0.01 | 0.01 |  | 0.01 | 0.01 | 0.02 |  | 0.01 | 0.01 | 0.01 |
|  |  |  |  |  |  |  |  |  |  |  |  |  |  |  |  |
| **σ[σ(XT)]** |  |  |  |  |  |  |  |  |  |  |  |  |  |  |  |
| Primary | 0.01 | 0.02 | 0.03 |  | 0.01 | 0.01 | 0.02 |  | 0.01 | 0.02 | 0.03 |  | 0.01 | 0.01 | 0.02 |
| Confirmatory | 0.02 | 0.03 | 0.04 |  | 0.01 | 0.02 | 0.03 |  | 0.02 | 0.03 | 0.04 |  | 0.02 | 0.02 | 0.03 |
| Summary | 0.02 | 0.04 | 0.06 |  | 0.02 | 0.03 | 0.05 |  | 0.03 | 0.03 | 0.06 |  | 0.02 | 0.03 | 0.05 |
| Other | 0.01 | 0.02 | 0.03 |  | 0.01 | 0.01 | 0.02 |  | 0.01 | 0.02 | 0.03 |  | 0.01 | 0.01 | 0.02 |
| Unspecified | 0.02 | 0.01 | 0.03 |  | 0.01 | 0.01 | 0.02 |  | 0.02 | 0.01 | 0.03 |  | 0.01 | 0.01 | 0.02 |
| All assays | 0.01 | 0.02 | 0.04 |  | 0.01 | 0.02 | 0.03 |  | 0.02 | 0.02 | 0.04 |  | 0.01 | 0.02 | 0.03 |

The overall average and standard deviation of the AID-specific average and standard deviation of the similarity score differences between the multiple-conformer model and single-conformer model approaches. “All assays” corresponds to all assays irrespective of type. “*Random*” corresponds to the 10-K set results from **Table 3**. “Single”, “All”, and “Best” correspond to search scenarios “A”, “B”, and “E” in **Table 1**, respectively.
